# Disruption of innate defense responses by endoglycosidase HPSE promotes cell survival

**DOI:** 10.1172/jci.insight.144255

**Published:** 2021-04-08

**Authors:** Alex Agelidis, Benjamin A. Turturice, Rahul K. Suryawanshi, Tejabhiram Yadavalli, Dinesh Jaishankar, Joshua Ames, James Hopkins, Lulia Koujah, Chandrashekhar D. Patil, Satvik R. Hadigal, Evan J. Kyzar, Anaamika Campeau, Jacob M. Wozniak, David J. Gonzalez, Israel Vlodavsky, Jin-ping Li, David L. Perkins, Patricia W. Finn, Deepak Shukla

**Affiliations:** 1 Department of Microbiology and Immunology,; 2 Department of Ophthalmology and Visual Sciences, and; 3 Division of Pulmonary, Critical Care, Sleep, and Allergy, Department of Medicine, University of Illinois at Chicago, Chicago, Illinois, USA.; 4 Department of Dermatology, Lurie Comprehensive Cancer Center, Northwestern University, Chicago, Illinois, USA.; 5 Department of Psychiatry, University of Illinois at Chicago, Chicago, Illinois, USA.; 6 Department of Pharmacology and; 7 Skaggs School of Pharmacy, UCSD, San Diego, La Jolla, California, USA.; 8 Technion Integrated Cancer Center (TICC), Rappaport Faculty of Medicine, Technion, Haifa, Israel.; 9 Department of Medical Biochemistry and Microbiology, University of Uppsala, Uppsala, Sweden.; 10 Division of Nephrology, Department of Medicine, and; 11 Department of Surgery, University of Illinois at Chicago, Chicago, Illinois, USA.

**Keywords:** Cell Biology, Microbiology, Cellular immune response, Innate immunity, Transcription

## Abstract

The drive to withstand environmental stresses and defend against invasion is a universal trait extant in all forms of life. While numerous canonical signaling cascades have been characterized in detail, it remains unclear how these pathways interface to generate coordinated responses to diverse stimuli. To dissect these connections, we followed heparanase (HPSE), a protein best known for its endoglycosidic activity at the extracellular matrix but recently recognized to drive various forms of late-stage disease through unknown mechanisms. Using herpes simplex virus-1 (HSV-1) infection as a model cellular perturbation, we demonstrate that HPSE acts beyond its established enzymatic role to restrict multiple forms of cell-intrinsic defense and facilitate host cell reprogramming by the invading pathogen. We reveal that cells devoid of HPSE are innately resistant to infection and counteract viral takeover through multiple amplified defense mechanisms. With a unique grasp of the fundamental processes of transcriptional regulation and cell death, HPSE represents a potent cellular intersection with broad therapeutic potential.

## Graphical abstract

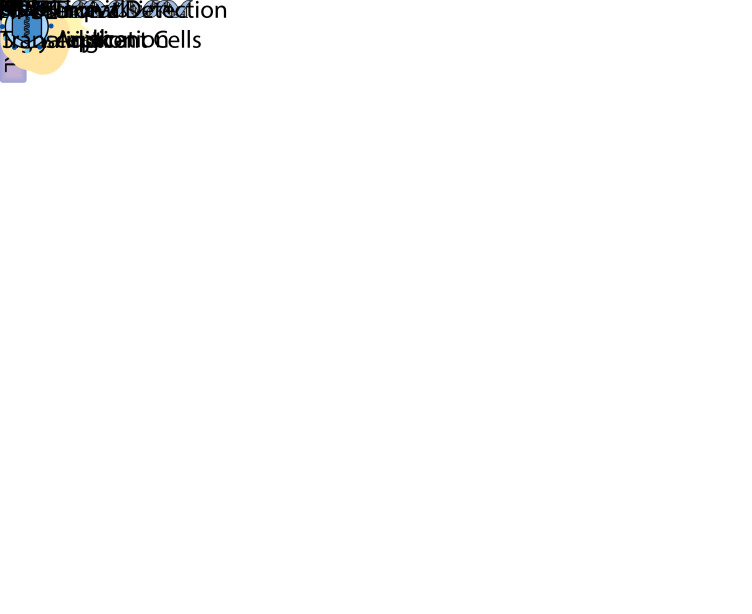

## Introduction

As major constituents of the extracellular matrix of virtually all cells, heparan sulfate and other protein-associated glycans have been well studied as coreceptors for a variety of macromolecules and pathogens. However, very little understanding of their regulatory mechanisms in cell signaling, microbial invasion, and cellular physiology exists. Some recent studies have noted that enzymatic splitting of these sugars at the plasma and nuclear membranes may constitute an important regulatory step that promotes inflammation and tissue invasion (1–3). An endoglycosidase, heparanase (HPSE), potentially contributes to pathological inflammatory signaling through its glycosidic action on the extracellular matrix. HPSE is the only known mammalian enzyme capable of splitting polymers of heparan sulfate, and heparan sulfate is the only known enzymatic target of HPSE (1). These chains of heparan sulfate hydrolyzed by HPSE may be present in multiple cellular locations in the context of various proteoglycans (4). However, HPSE remains a mysterious cellular component, as additional reports of its tendency to appear in the nucleus and regulate gene expression continue to emerge (5, 6). Associations between HPSE and various pathologies, including inflammation and cancer metastasis, have historically been attributed to the protein’s cleavage of HS chains at the cell surface and basement membrane (1, 2); however, HPSE may possess unique roles arising independently of its enzymatic active site. Recently, our group and others have described yet another important role for HPSE as a driver of microbial pathogenesis. HPSE is now known to be transcriptionally upregulated by several viruses dependent upon heparan sulfate for attachment and entry, and subsequently facilitates egress of newly produced viral particles (7–10). While enzymatic activity of HPSE is believed to enable viral release through splitting of HS residues at the cell surface, it has been reported to also regulate gene expression and trigger proviral signaling through some distinct nonenzymatic activity (11). With a focus on cellular responses to infection, we show here that HPSE acts beyond its known endoglycosidase activity as a potent regulator of the signal transduction phase of cellular defense.

## Results

### Cells lacking HPSE are intrinsically resistant to HSV-1 infection.

**Figure 1:**
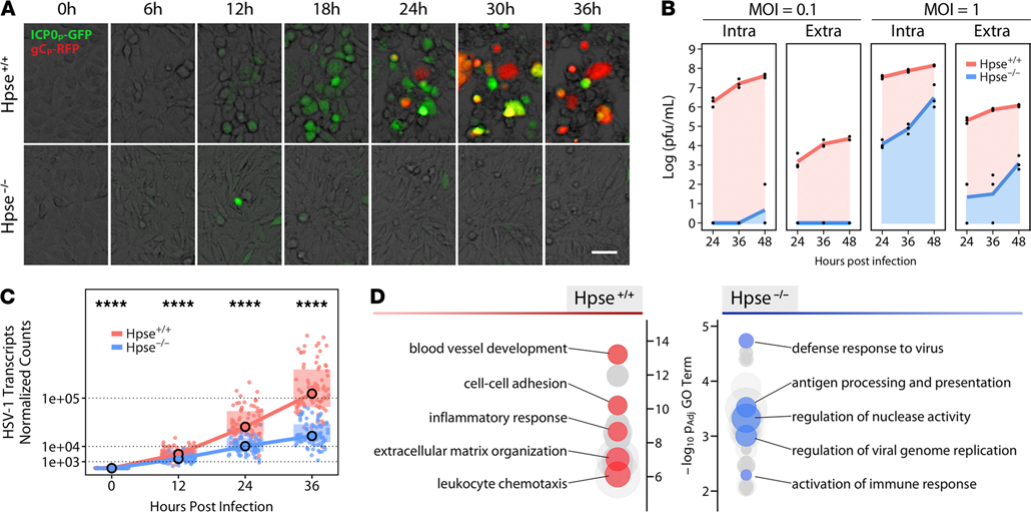
Baseline differences in host gene expression grant HPSE-deficient cells resistance to viral infection. (**A**) Representative images of infection time course of WT (Hpse^+/+^) and heparanase-deficient (Hpse^-/-^) mouse embryonic fibroblasts (MEFs) with a dual fluorescence strain of HSV-1. Green signal indicates viral immediate early gene expression (ICP0), while red signal indicates viral late gene expression (gC). Scale bar: 50 μm. (**B**) Titers of intracellular (Intra) and extracellular (Extra) HSV-1 detected at 2 multiplicities of infection (MOI) in WT and Hpse^-/-^ MEFs (*n* = 3). (**C**) HSV-1 viral gene expression as quantified by transcriptomics analysis. Each data point represents 1 of 74 detected viral transcripts. Median and IQR are plotted; significance analyzed by Wilcoxon signed-rank test. (**D**) Baseline differential enrichment of gene ontology terms of Hpse^+/+^ and Hpse^-/-^ cells in the absence of infection. Each node represents a unique term, with node size representing fold enrichment. Select summary terms are highlighted. ****P < 0.0001.

Given our unexpected finding that HPSE can directly drive viral pathogenesis (7, 11), we became interested in investigating cellular responses to infection in the absence of HPSE altogether. To our further surprise, HSV-1 infection of Hpse-KO mouse embryonic fibroblasts (MEFs) showed a dramatic reduction in virus production, as compared with WT MEFs. Virus enters cells at normal levels, but viral replication and protein production are decreased by several orders of magnitude (Figure 1, Supplemental Figure 1, and Supplemental Tables 1–5; supplemental material available online with this article; https://doi.org/10.1172/jci.insight.144255DS1). Hpse-KO cells originally appear capable of immediate early viral gene production, but late gene products are effectively absent (Figure 1A). These findings align with previous work from our group and others showing that active HPSE translocates to the nucleus and influences gene expression through an unknown mechanism (5, 6, 12–14). We therefore adopted an unbiased approach to generate a clearer understanding of how HPSE, or a lack thereof, potently influences gene expression and cell signaling to restrict viral production. RNA sequencing (RNA-seq) analysis constituted the primary exploratory and gene clustering pipeline, while quantitative proteomics analysis of the same samples provided an additional level of functional validation. The transcriptomic landscape of Hpse-deficient cells is markedly altered by ablation of this single locus, with 1385 genes showing baseline differences in expression (Supplemental Figure 1). Based on gene ontology (GO) analysis of noninfected cells, we observed that Hpse-deficient cells show significant enrichments of genes representing pathways of “defense response to virus” and “activation of immune response,” suggesting that these cells are somehow intrinsically resistant to infection (Figure 1D). As an independent confirmation, differential expression analysis of the only existing publicly available data set of HPSE genetic alteration (GSE34080) yielded strikingly similar results (Supplemental Figure 2). This silencing RNA knockdown of HPSE performed by other investigators in human gastric cancer cells shows significant enrichment of GO terms, including “negative regulation of viral genome replication” and “response to type I interferon,” indicating a broad external validity of these newly described HPSE functions.

### Temporal viromics catalog transcriptional landscape shifts dependent on HPSE.

**Figure 2:**
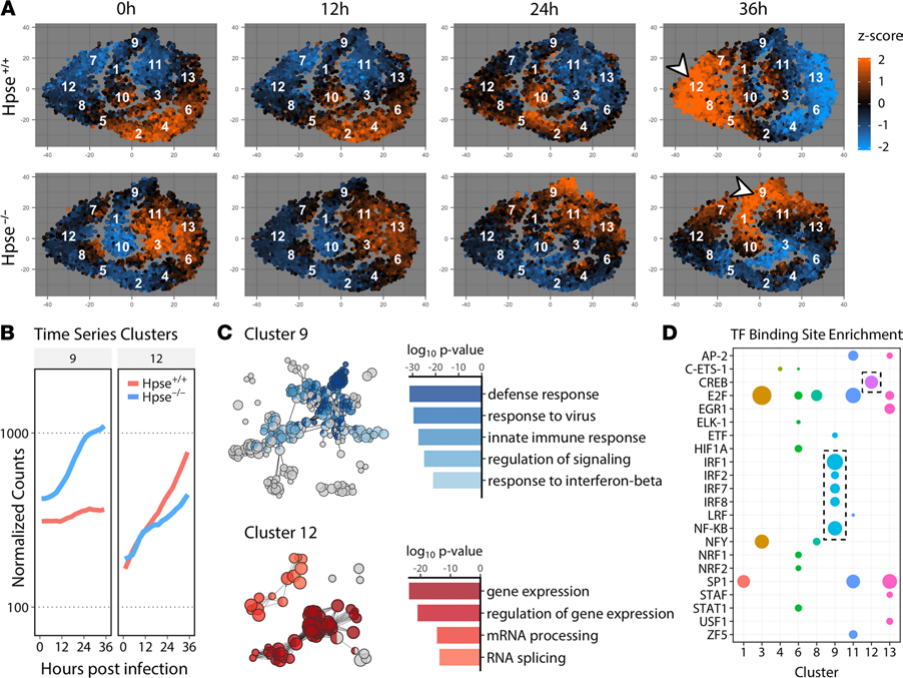
Temporal viromics catalogs shift in transcriptional landscape and reveal functional clusters of genes dependent on HPSE. (**A**) t-Stochastic neighbor embedding (t-SNE) projection depicting relative expression of differentially expressed genes, with clustering based on expression patterns over infection time course. Centroids of each of 13 clusters are labeled with respective identifier. Arrowheads indicate clusters further dissected below. (**B**) Median gene expression of selected clusters displayed over time. (**C**) Representation of significantly enriched gene ontology (GO) terms performed with ClueGO. Node sizes relative to GO term enrichment, with functionally related terms grouped by color and indicated by corresponding bar plot. (**D**) Significant transcription factor (TF) binding site enrichment analysis represented by cluster, with bubble sizes relative to enrichment score.

Hundreds of gene expression changes occur in a viral infection, and as such, viruses represent excellent tools to probe the connections between cellular processes and signaling cascades. To appreciate major regulators of this cellular remodeling and understand how these shifts are influenced by HPSE, we generated clusters of the significantly differentially expressed genes based on temporal expression patterns. Topological clustering using a t-distributed stochastic neighbor embedding (t-SNE) (15) followed by an affinity propagation (APcluster) (16) algorithm produced 13 well-defined clusters (Figure 2A and Supplemental Figures 3 and 4). Further attention to the most heavily induced clusters 9 and 12 revealed that Hpse-deficient cells are hyperactive in antiviral cytokine signaling and cellular defense, while infection of WT cells stimulates production of cellular machinery essential for virus assembly (Figure 2, B and C). In fact, cluster 12 also contains all viral genes, which are transcribed virtually unimpeded in WT cells, while expression of this cluster is stalled in Hpse-KO, concordant with the defense response induction occurring around 12 hours postinfection (hpi). We then scanned upstream promoter regions of genes in each cluster using the PASTAA algorithm to identify major transcriptional regulators and correlate with temporal expression patterns (Figure 2D). Our finding of highly significant enrichments of multiple IFN regulatory factors (IRFs) and NF-κB as the primary regulators of cluster 9 expression adds further credence to results of the above GO analysis and previous in vitro evidence of an inverse relationship between IRF7 and HPSE gene expression (11). Interestingly, this analysis also designated cAMP response element binding protein (CREB) as a major proviral factor, and remarkably, pharmacological blockade of various components of the CREB signaling interface resulted in potent inhibition of virus production in both WT MEFs and human epithelial cells. These results further express the value of mechanistic characterization and temporal dissection of gene expression shifts in driving rational drug discovery.

### HPSE restricts direct antimicrobial activity of type I IFN system.

**Figure 3:**
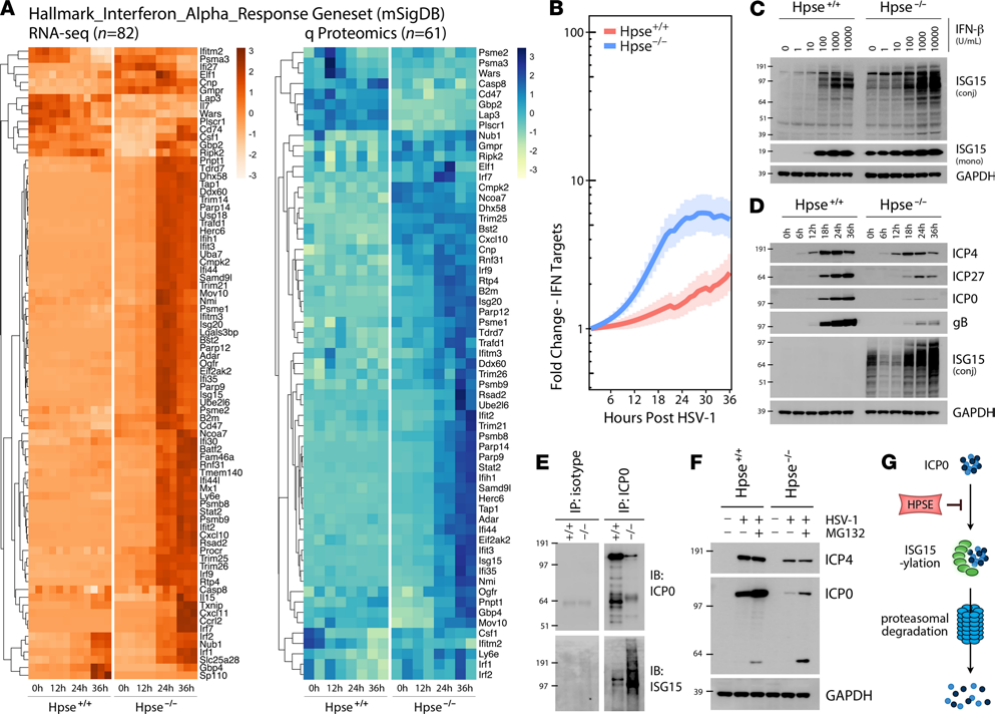
HPSE restricts type I IFN response and drives virus production through inhibition of degradation of immediate early protein ICP0. (**A**) Transcriptomic and proteomic datasets filtered based on Hallmark_Interferon_Alpha_Response gene set (*n* = 91 genes) obtained from mSigDb. (**B**) Transcript expression of Hallmark_Interferon_Alpha_Response subset displayed as fold change over baseline, with bolded lines signifying mean expression and shaded areas indicating SEM. Splines generated using MetaLonDA. (**C**) Increased type I IFN sensitivity in Hpse^-/-^ cells at baseline and with IFN-β administration for 18 hours, exemplified by expression of IFN-stimulated gene (ISG15) monomer and protein-conjugated forms. (**D**) Representative Western blot analysis of cell lysates from WT and Hpse^-/-^ MEFs indicating selective defect in viral protein production in HPSE deficiency. gB is a late (γ) gene, while ICP4, ICP27, and ICP0 are immediate early (α) genes. (**E**) Western blot analysis of cell lysate, isotype immunopurification, and ICP0 immunopurification of WT and Hpse^-/-^ cells after 24-hour HSV-1 infection, with 10 μM MG132 added for the final 4 hours. (**F**) Immediate early viral protein expression in the presence of proteasome inhibitor MG132 (10 μM for last 4 hours of 24-hour infection) shown by Western blot analysis of cell lysates. (**G**) Proposed model depicting ability of HPSE to interfere with ISG15 conjugation and proteasomal degradation of viral ICP0.

Given the amplified induction of defense and immune-related genes observed in our unbiased analysis of Hpse-deficient cells, we sought to mechanistically define the apparent link between HPSE and innate immune signaling. Subsetting both transcriptomics and proteomics data sets based on the curated Hallmark_Interferon_Alpha_Response gene set (MSigDB; http://www.gsea-msigdb.org/gsea/msigdb/index.jsp) shows the striking upregulation of IFN-stimulated genes (ISGs) in the absence of HPSE (Figure 3, A and B). IFNs represent a highly conserved family of cytokines, with type I IFN (α and β) secreted particularly in response to viral infection and known to exert various antimicrobial functions (17). Using ISG15 as an indicator of signaling activity downstream of type I IFN, Hpse-KO cells are observed to be intrinsically more sensitive to these conserved cytokines. In the absence of HPSE, cells are responsive to levels of purified IFN-β orders of magnitude lower than WT cells, with active signaling occurring even without any external stimulus (Figure 3C). Interestingly, with a similar size and structure to that of ubiquitin, ISG15 is known to exert antimicrobial activity through direct protein ligation, with downstream consequences including proteasomal degradation and regulation of signal transduction (18). Expression analysis of individual viral proteins by proteomics analysis showed that Hpse-KO cells exhibit a particularly large defect in production of the immediate early protein ICP0, which is known to be essential for HSV-1 replication and assembly (Figure 3, D–F, and Supplemental Figure 5) (19, 20). Blocking of the proteasome with MG132 for the last 4 hours of infection somewhat restores ICP0 (110 kDa) and also reveals increased levels of a 66 kDa degradation product previously observed by other investigators (21). Our findings here detail a mechanism of direct antimicrobial action occurring in the absence of HPSE: conjugation of ISG15 to ICP0 correlates with increased proteasomal degradation of this essential viral protein, thus stalling viral replication (Figure 3G).

### Deletion of HPSE protects against cellular infiltration and associated inflammation.

**Figure 4:**
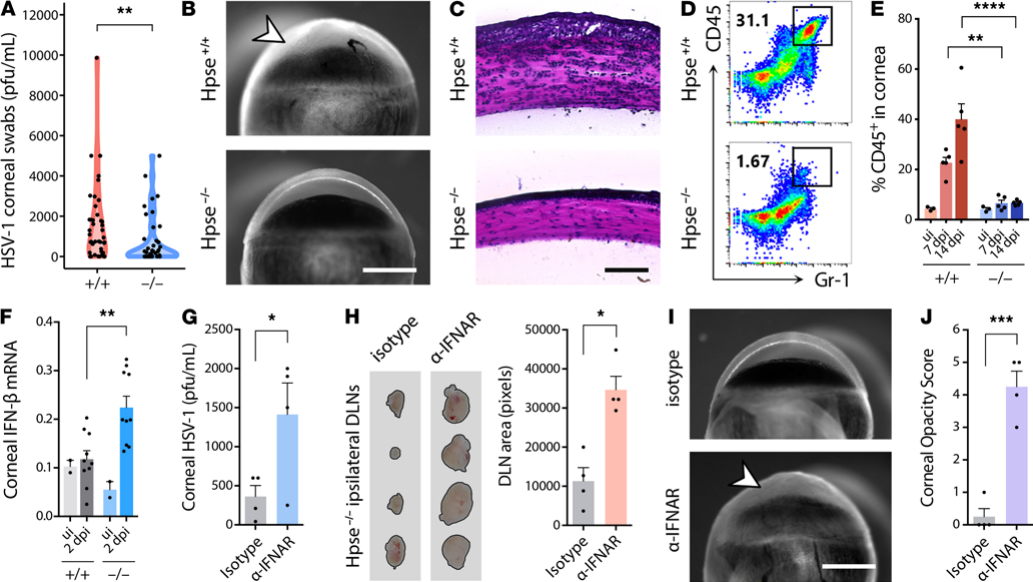
Deletion of HPSE protects against cellular infiltration and associated inflammation in a murine model of corneal infection. (**A**) HSV-1 titers 2 days following corneal infection of Hpse^+/+^ and Hpse^-/-^ mice (*n* = 44). Significance determined by Wilcoxon signed-rank test. (**B**–**D**) Markedly decreased corneal inflammation and infiltration (arrowhead) in Hpse-deficient mice 14 days following HSV-1 infection observed by gross imaging (**B**), H&E histology (**C**), and flow cytometry of dissociated corneal tissues (**D**). Scale bars: 1 mm (**B**), 50 μm (**C**). (**E**) Quantification of leukocytes (CD45^+^) present in corneal tissue at indicated dpi as observed by flow cytometry (*n* = 5 for infected animals, *n* = 3 for uninfected animals). Significance determined by 2-way ANOVA with Sidak correction for multiple comparisons. (**F**) Corneal IFN-β mRNA copy number relative to β-actin measured at 2 dpi (*n* = 10). Uninfected (ui) samples constituted 2 pools of 5 mouse corneas each. Significance determined by 2-way ANOVA with Sidak correction for multiple comparisons. (**G**–**J**) Partial restoration of viral titers and cellular infiltration in HPSE-deficient mice after topical application of α-IFNAR monoclonal antibody, observed by ocular wash titers (**G**), gross analysis of ipsilateral draining lymph nodes (DLN) (**H**), and gross analysis (**I**) and scoring (**J**) of corneal opacity (*n* = 4). Scale bar: 1 mm. Data represent mean ± SEM. Significance determined by unpaired *t* test unless otherwise specified. *P < 0.05, **P < 0.01, ***P < 0.001, ****P < 0.0001.

To appreciate a broader impact of these findings, we evaluated infection in Hpse-KO mice (22), which showed a significant decrease in virus production by plaque assay after corneal HSV-1 infection (Figure 4A). Despite the more modest reduction in virus titers compared with observations in vitro, HSV-1–infected corneas displayed a striking ablation of the typical inflammatory response observed in WT mice, evidenced by loss of CD45^+^Gr-1^+^ neutrophil and monocyte infiltration at 7 and 14 days postinfection (dpi) (Figure 4, B–E). Absolute cell infiltration counts and flow cytometry gating strategy are displayed in Supplemental Figure 6. In accordance with previous studies of the murine keratitis model, we observed that viral titers peak in both genotypes at 2 dpi, while substantial cellular infiltration to the cornea does not occur before 5 dpi and was, thus, measured at 7 and 14 dpi (23, 24). Infected Hpse-KO mice also showed a significant increase in corneal IFN-β mRNA, in line with our in vitro findings (Figure 4F). A baseline difference in IFN-β transcripts was not observed in corneal epithelium, possibly due to additional compensatory changes that occurred in the process of cell differentiation in vivo. Furthermore, ocular application of monoclonal antibodies against IFN-α receptor (α-IFNAR) resulted in a significant restoration of virus production and infiltrative phenotypes, including increased cellularity of draining lymph nodes and marked corneal opacity (Figure 4, G–J). These results may suggest that resident corneal cells lacking HPSE are intrinsically more effective in neutralizing the virus rather than inciting the nonspecific granulocytic infiltration known to be pathogenic in herpes keratitis. Collectively, these findings demonstrate that HPSE restriction of the type I IFN system is a potent immunomodulatory mechanism, and upregulation of HPSE may be a common microbial strategy for evasion of innate immune responses.

### Necroptosis is an IFN-induced stress response limited by HPSE.

**Figure 5:**
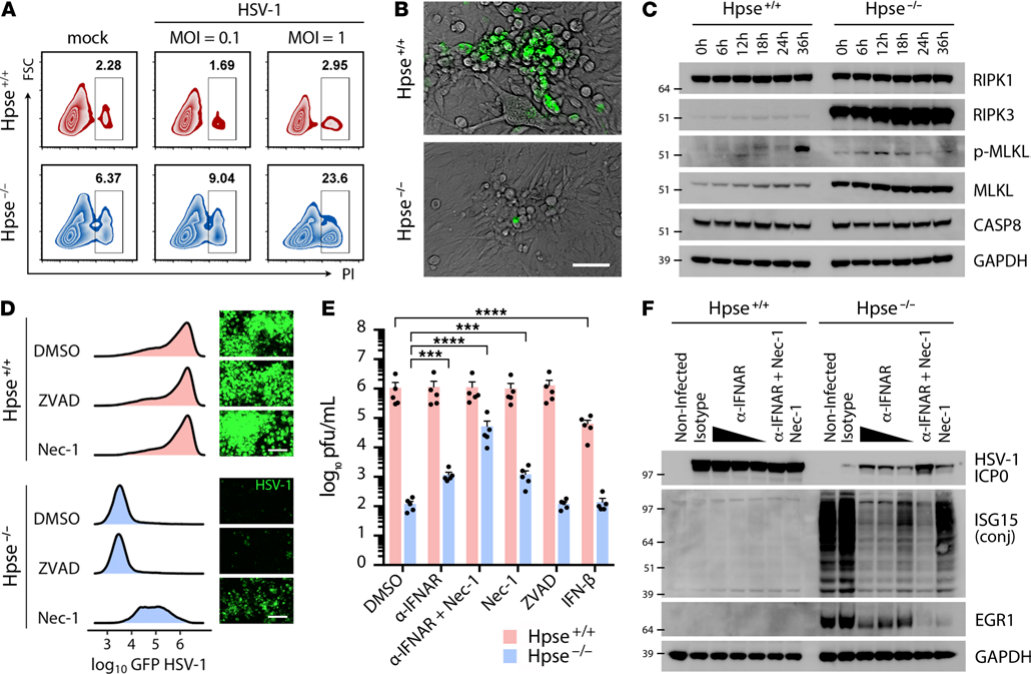
Attenuation of IFN response and necroptotic cell death restores infection in the absence of HPSE. (**A**) Measurement of cell death by flow cytometry detection of propidium iodide (PI) cellular uptake after 24-hour HSV-1 or mock infection. (**B**) Immunofluorescence microscopy of cells infected with GFP–HSV-1; images captured at 24 hpi. Despite profound abrogation of virus production in the absence of HPSE, multiple clusters of rounded and detached cells resembling plaques are observed after infection. Scale bar: 50 μm. (**C**) Western blot analysis of key proteins involved in induction of necroptosis at indicated times after infection. (**D**) Representative flow cytometry quantification and micrographs of Hpse^+/+^ and Hpse^-/-^ cells after infection with GFP–HSV-1 for 48 hours, incubated with inhibitors of apoptosis (ZVAD) or necroptosis (Nec-1) as indicated. Scale bar: 100 μm. (**E**) Restoration of virus production in Hpse^-/-^ cells with blocking of IFN-α receptor (α-IFNAR) and necroptosis (Nec-1) showing a synergistic effect (*n* = 5). (**F**) Inverse relationship between viral infected cell protein (ICP0) and ISG15 expression demonstrated by Western blot, with near complete rescue of virus production observed upon α-IFNAR and Nec-1 treatment in Hpse^-/-^ cells. Data represent mean ± SEM. Significance determined by 2-way ANOVA with Dunnett’s correction for multiple comparisons against control (DMSO). ***P < 0.001, ****P < 0.0001.

Another initial observation from our studies was that Hpse-deficient cells appear to possess the intrinsic ability to contain infection before considerable viral spread can occur. This finding suggests that, in the absence of HPSE, infected cells undergo some form of programmed cell death to shut down virus production. Indeed, Hpse-KO cells are more prone to virus-induced cell death and possibly exhibit increased levels of basal cell death, measured as increased PI uptake by flow cytometry (Figure 5A). Although effective viral production is markedly hindered in the absence of HPSE, small clusters of rounded and dead cells similar to plaques are frequently observed after infection (Figure 5B). Interestingly, recent studies have suggested that necroptosis, or programmed necrosis, is a major mechanism of virus-induced cell death, which may provide some level of protection to the infected host (25–27). Moreover, type I IFNs are known drivers of necroptosis in response to various cellular stresses including viral infection (17). Western blot analysis of an infection time course showed a pronouncedly increased abundance of a key protein required for induction of necroptosis, receptor-interacting serine/threonine-protein kinase 3 (RIPK3), in the absence of HPSE (Figure 5C). Increased abundance of mixed lineage kinase domain–like pseudokinase (MLKL) was also observed, while no apparent difference in other key proteins including RIPK1 and caspase-8 was noted. Phosphorylated MLKL (p-MLKL), an indication of necroptosis activation, was found to be elevated at baseline in Hpse-KO cells and induced as early as 12 hpi, whereas WT cells did not show any considerable level of p-MLKL until 36 hpi. This finding suggests that HPSE-deficient cells undergo some basal level of necroptosis and that these cells are more sensitive to necroptosis induction by exogenous stimuli. Using inhibitors of the type I IFN system (α-IFNAR) and necroptosis (necrostatin-1; Nec-1) in combination, infection in Hpse-KO cells was nearly restored to WT levels (Figure 5, D–F). Evaluation of inhibitors of apoptosis (ZVAD) and necroptosis (Nec-1) shows that HPSE preferentially limits necroptosis to promote cell survival and, thus, virus production. Since many viruses, including HSV-1, are known to block cell death to promote viral production and spread, clearance of infected cells through HPSE inhibition may hold great therapeutic potential.

### Bioinformatics-guided analysis of transcription factor activation in HSV-1 infection identifies potent antiviral compounds.

**Figure 6:**
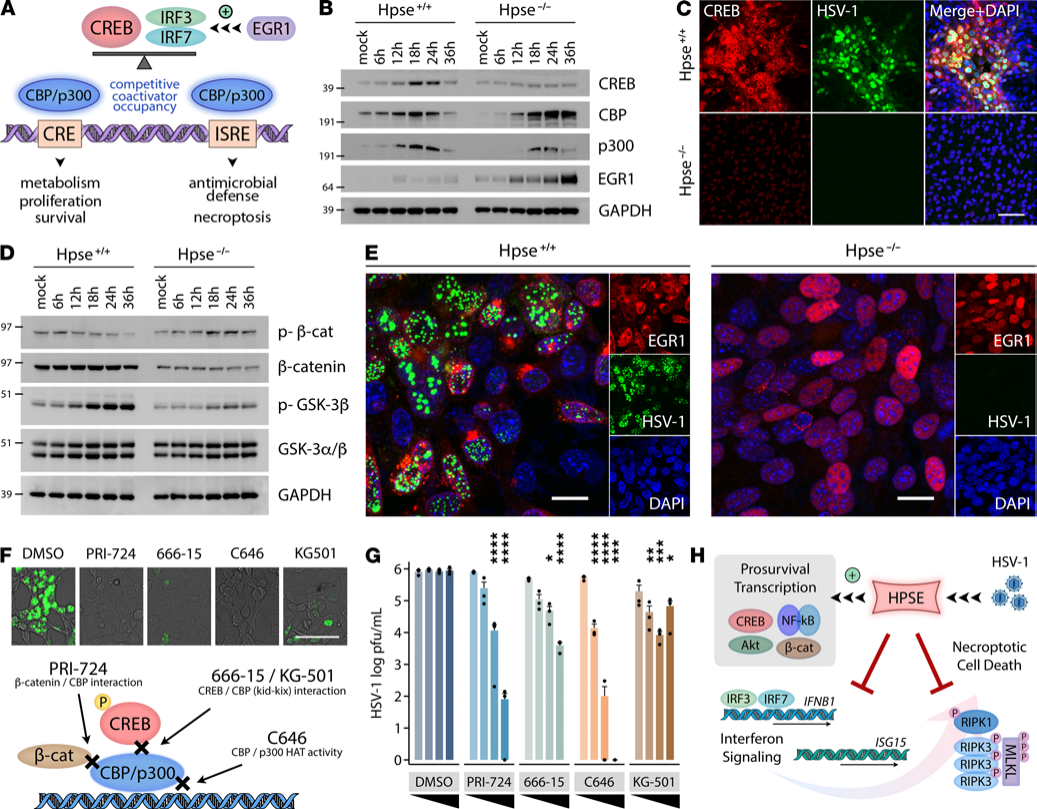
Bioinformatics-guided analysis of transcription factor activation in viral infection identifies potent antiviral compounds. (**A**) Schematic depicting CREB and IRF competitive binding for CBP/p300 transcriptional coactivators, based on published literature (28, 29, 32, 33). (**B**) Representative Western blot analysis of CREB signaling induction with infection of WT and Hpse-KO cells. (**C**) Confocal immunofluorescence microscopy of WT and Hpse-KO cells showing CREB upregulation in infected WT cells. Scale bar: 100 μm. (**D**) Representative Western blot analysis of β-catenin signaling induction with infection of WT and Hpse-KO cells. (**E**) Confocal immunofluorescence microscopy of WT and Hpse-KO cells showing EGR1 cellular localization and GFP–HSV-1. Scale bar: 20 μm. (**F**) Top: Representative immunofluorescence micrographs of human corneal epithelial cells infected with GFP–HSV-1 and then incubated with specified inhibitors at 2 hours after GFP–HSV-1 infection; images captured at 24 hpi. Scale bar: 100 μm. Bottom: Schematic depicting known mechanisms of action of selected inhibitors. (**G**) Viral titers obtained from human corneal epithelial cells after incubation of specified inhibitors at the following concentrations from left to right: 12.5, 25, 50, and 100 µM (666-15 was used at concentrations of 5, 10, 15, and 20 μM due to unacceptable toxicity at higher levels). Significance determined by 2-way ANOVA with Dunnett’s correction for multiple comparisons against control (DMSO) at respective concentrations (*n* = 3). (**H**) Model of HPSE function at the interface of innate defense responses and cell survival. Infection and other cellular insults trigger activation of multiple prosurvival factors, including CREB, Akt, NF-κB, and β-catenin. Previous work and this study show that HPSE modulates nuclear trafficking of these transcription factors (TFs), which drive cellular proliferation, microbial replication, and carcinogenesis. Here, we show that HPSE inhibits type I IFN production and induction of necroptosis. These innate stress responses likely act to protect multicellular tissues from viral or cancerous spread by preventing uncontrolled cellular proliferation.

While gaining an understanding of the major antiviral pathways active in Hpse-KO cells, we remained interested in targeting proviral networks activated upon infection of WT cells. Analysis of divergent responses between these 2 cell types provided the initial clues that CREB and β-catenin are drivers of proviral signaling. CREB was identified as a key driver of cluster 12 expression through transcription factor binding site analysis of RNA-seq data (Figure 2), while β-catenin was detected as a major hub gene from proteomics analysis of differentially expressed proteins. In light of these results and the above findings that HPSE inhibits functional IFN signaling, we pursued further experiments under the following rationale (Figure 6A). Years of published work informed us that IRFs, including IRF3, IRF7 and IRF9, bind to CREB-binding protein (CBP) and histone acetyltransferase p300 to drive optimal transcription of type I IFN and downstream IFN-stimulated genes (28, 29). Thus, these IRFs, in effect, compete with CREB and other transcription factors for CBP/p300 occupancy and binding to respective cAMP response elements (CRE) or IFN-sensitive response elements (ISRE). We also found that an inverse association exists between the CREB and IFN in that inhibitors of the CREB/CBP/p300 system resulted in induction of IFN-β in human cells (Supplemental Figure 7A). Conversely, exogenous IFN-β led to a decrease in total CREB protein levels in cells lacking HPSE (Supplemental Figure 7B). Additionally, we focused some attention to EGR1, a transcription factor shown by multiple studies to drive HPSE upregulation (30, 31) and to act as a supporter of IFN signaling (32, 33). Interestingly, various studies have also drawn connections of EGR1 with necroptosis, CREB signaling, and viral infection (34–37).

Indeed, Western blot and immunofluorescence microscopy confirmed that activation of the CREB and β-catenin systems occurs upon infection of WT MEFs, while Hpse-KO cells do not display these changes (Figure 6, B–D). CREB is upregulated in WT cells starting at 12 hpi, while this response is absent in Hpse-KO. Likewise, phosphorylation of β-catenin at the S33/S37/T41 sites, indicative of inactive β-catenin (38), is decreased across infection time points, while this phosphorylation is increased in Hpse-KO. A similar trend is observed for p–GSK-3β, which when phosphorylated at the S9 site is known to result in β-catenin activation through double inhibition (38). We also noted profound differences in EGR1 abundance and localization dependent on HPSE expression (Figure 6E). Given these supportive findings, we opted to treat cells with inhibitors targeting the CREB or β-catenin systems by therapeutic application at various concentrations at 2 hpi after viral entry had occurred. The identities and mechanisms of these compounds are shown in Figure 6F. Initial testing was performed in WT MEFs; upon detection of antiviral efficacy, we transitioned to human corneal epithelial (HCE) cells, a model cell line for HSV-1 infection. Images from GFP–HSV-1 and viral titers from HCE cell culture supernatants collected at 24 hpi demonstrate antiviral efficacy of these compounds (Figure 6, F and G). By merging our unbiased temporal analysis with various molecular manipulations, we show that, through activation of these 2 host factors with broad control over cellular physiology, HSV-1 generates an environment conducive to its own propagation and persistence (Figure 6H).

## Discussion

With this multidisciplinary exploration of various cellular responses to stress, we demonstrate how HPSE serves as a key intersection between the detection and effector phases of signal transduction. Using a genome-wide analysis of HPSE-deficient cells, we aimed to define the factors and related pathways that make these cells naturally resistant to infection. Our results show that considerable differences in baseline gene expression exist in these cells compared with WT, possibly due to compensatory mechanisms that have emerged throughout embryonic development in the absence of HPSE. Viral infection triggers extensive remodeling of host cellular processes. By understanding the connections between these processes, we can infer their reliance on HPSE in the case of WT cells. The temporal viromics methods employed here reliably clustered differentially expressed genes and identified transcription factors regulating major shifts in cellular programs over time. This analysis draws an indirect connection between HPSE and multiple important drivers of cellular proliferation and defense. In agreement with our previous work, we show by transcription factor binding site analysis that IRFs and NF-κB are major drivers of the defense response gene cluster heavily upregulated with infection in Hpse-KO cells. We further dissect this relationship in vitro and in vivo by rescuing virus production in Hpse-KO with inhibition of the IFN and necroptosis systems. Of note, a majority of studies has used multiplicities of infection (MOI) of HSV-1 in the 1–100 range for induction of IFN signaling in MEFs. In this study, we focused on an MOI of 0.1, more commensurate to productive infection occurring in vivo. Our results highlight the increased sensitivity of Hpse-KO cells to induction of the type I IFN response upon low-grade viral infection. Future studies will aim to more precisely define the nature of the first-order interactions between HPSE and effectors, as these remain almost entirely unknown. Given the regulatory relationship that exists between EGR1 and HPSE (30, 31), we suspect that in the absence of HPSE expression, EGR1 upregulation upon infection remains unchecked and that this uncontrolled EGR1 level results in hyperactivation of the type I IFN system. Further mechanistic studies will be required to dissect the antiviral mechanisms displayed by EGR1. Moving forward, high-throughput analyses with even greater temporal resolution will continue to unearth unique connections not previously imagined, yet the importance of molecular validation by conventional methods will remain. Additionally, these newly reported activities of HPSE can help explain the documented roles of HPSE in cancer progression. Numerous malignancies have been shown to increase expression of HPSE, understood to drive late-stage metastatic potential (1, 2). Recent studies have also shown that the type I IFN system has control over cancer spread as depletion of IRF7 increases metastatic burden in mice and humans (39, 40).

In search for the mechanism of cell death occurring preferentially in cells lacking HPSE, we considered contributions from multiple cellular systems including reactive oxygen species (ROS), mitophagy, inflammasome activation, pyroptosis, and apoptosis. We observed no difference in ROS production occurring between genotypes or upon infection; however, ROS scavenging compounds like N-acetylcysteine and urolithin A were indeed found to decrease ROS production as expected (Supplemental Figure 8). Induction of mitophagy with urolithin A does appear to have an alleviating effect on cell death in Hpse-KO cells as observed by propidium iodide uptake. Gene subset analysis of our transcriptomics and proteomics data suggested that some differences exist in mitophagy baseline levels and induction, dependent on HPSE; however, no clear expression pattern or gene candidate stood out at this time, and this will be the subject of ongoing studies. On the other hand, p-MLKL appears much earlier in infection in Hpse-KO cells, suggesting that this enhanced initial sensitivity is important in the marked difference in the subsequent host cell response. A more rapid induction of p-MLKL and substantial rescue of virus production with Nec-1 occurring in HPSE-deficient cells point toward necroptosis as a major form of cell death occurring in the absence of HPSE. Since ZVAD acts by blocking the active site of caspases, the fact that virus production is partially restored with Nec-1 and not ZVAD suggests that a caspase-independent form of cell death is dominant. Furthermore, Western blot analysis showed negligible differences in inflammasome and pyroptosis activation (noted by IL-1β, caspase-1, and gasdermin D cleavage) and only minor levels of apoptosis activation occurring at later time points (noted by caspase-3 and caspase-8 cleavage) (Supplemental Figure 9). Additionally, no apparent difference in accumulation of ubiquitinated proteins was observed between the 2 cell types. Full-length Western blots for all figures are presented in Supplemental Figure 10.

We also identify 2 potentially novel proviral transcriptional regulatory factors CREB and β-catenin that are activated in WT cells, and we demonstrate that their pharmacological inhibition in human cells is a highly effective antiviral strategy resulting from this analysis. We show that, through activation of these 2 factors with broad control over cellular physiology, the virus generates an environment conducive to its own success. CREB is a transcription factor well studied in the neuroscience field but with little mention in studies of microbial pathogenesis. It is known to be activated by protein kinases upon second messenger signaling and bound by coactivators CBP and p300 for its binding to DNA sequences (41). CREB has also been documented to interact with various signaling molecules, including signal transducer and activator of transcription 1 (STAT1) and NF-κB, indicating potential ramifications for generation of immune responses (42, 43). Likewise, β-catenin is a well-studied regulator of transcription and cell adhesion overexpressed in several cancers, and it is part of a multiprotein destruction complex. Numerous protein interactions and regulators of β-catenin have been characterized, with one known transcriptional coactivator being CBP for DNA binding. The association between this prosurvival factor and infection remains understudied, since only a small number of studies exist linking HSV-1 or other viral infection to β-catenin activation (44–46). In this study, we show that multiple commercially available compounds targeting CREB and β-catenin systems demonstrate antiviral efficacy against HSV-1 and display an inverse relationship with type I IFN signaling. While some degree of cellular toxicity was noted at higher concentrations, all compounds tested displayed antiviral efficacy, with PRI-724 showing the most appealing profile of antiviral activity over toxicity. Interestingly, this compound is currently in clinical trials for hepatitis C virus hepatocellular carcinoma and other cancers (47); along with the other compounds analyzed here, it may prove a promising antiviral candidate. Our findings showcase a success of mechanism-based rational drug design informed by multiperspective analysis merging systems and molecular investigation.

## Methods

### Mice.

6- to 10-week-old male and female mice on C57BL/6 background (WT or Hpse-deficient) were used for all experiments. Anesthetized mouse corneas were scarified in a 3 × 3 grid using a 30-gauge needle and infected as previously described (11). All images of the corneal surface were acquired with SteREO Discovery.V20 stereoscope (Zeiss). For IFNAR blockade experiment, 5 μL α-IFNAR monoclonal antibody (Leinco I-401) was applied topically to corneas of mice once 24 hours prior to infection and then daily for 3 days after infection. Ocular infection was performed as described above, and mice were sacrificed at 45 dpi for tissue analysis.

### Cell lines and virus strains.

HCE cell line (RCB1834 HCE-T) was obtained from Kozaburo Hayashi (National Eye Institute, Bethesda, Maryland, USA) and was cultured in MEM (Invitrogen) with 10% FBS (Invitrogen) and 1% penicillin/streptomycin (Invitrogen). Confirmation of the identity of HCE cell line was done by short tandem repeat analysis. Vero cell line for virus preparation and plaque assay was provided by Patricia G. Spear (Northwestern University) and cultured in DMEM (Invitrogen) with 10% FBS and 1% penicillin/streptomycin. WT and Hpse-KO MEFs were provided by Israel Vlodavsky (Rappaport Institute, Haifa, Israel) (22). All cells were maintained in a Heracell VIOS 160i CO_2_ incubator (Thermo Fisher Scientific) and have been confirmed negative for mycoplasma contamination. Virus strains used in these studies, HSV-1 (KOS-WT) (48), GFP–HSV-1 (K26-GFP) (48), HSV-1 (gL86) (49), McKrae, and pseudorabies virus (PRV) (50) were provided by Patricia G. Spear (Northwestern University, Chicago, Illinois, USA). Dual-color ICP0_p_-GFP/gC_p_-RFP was a gift from Paul Kinchington (University of Pittsburgh, Pittsburgh, Pennsylvania, USA). HSV-1 infections were performed with strain KOS at a MOI of 0.1 unless otherwise specified. All virus stocks were propagated in Vero cells and stored at –80°C.

### Antibodies and plasmids.

For Western blot studies, the following antibodies from Cell Signaling Technology were used, at dilution 1:1000: CREB (catalog 9197), CBP (catalog 7389), p300 (catalog 86377), β-catenin (catalog 8480), phospho–β-catenin (catalog 9561), EGR1 (catalog 4154), RIPK1 (catalog 3493), RIPK3 (catalog 95702), MLKL (catalog 37705), phospho-MLKL (catalog 37333), CASP1 (catalog 24232), cleaved CASP1 (catalog 89332), CASP3 (catalog 14220), cleaved CASP3 (catalog 9664), CASP8 (catalog 4927), IL-1β (catalog 12507), cleaved IL-1β (catalog 63124), gasdermin D (catalog 39754), and cleaved gasdermin D (catalog 10137). From Santa Cruz Biotechnology Inc., at dilution 1:250, the following antibodies were used: GSK-3β (catalog sc-7291), phospho–GSK-3β (catalog sc-373800), ICP0 (catalog sc-53070), ICP4 (catalog sc-69809), ICP27 (catalog sc-69806), ISG15 (catalog sc-166755), and ubiquitin (catalog sc-8017). From Proteintech, at dilution 1:1000, anti-GAPDH (catalog 10494) was used, and from Abcam, at dilution 1:10,000, anti-gB (catalog ab6505) was used. For immunofluorescence microscopy studies, the following antibodies from Cell Signaling Technology were used, at dilution 1:100: CREB (catalog 9197) and EGR1 (catalog 4154). HPSE expression constructs including Myc-GS3 plasmid were provided by Israel Vlodavsky (Rappaport Institute) (51). Lipofectamine-2000 transfection reagent (Invitrogen, 11668019) was used for all in vitro overexpression experiments, according to the manufacturer’s specifications.

### Chemical reagents.

Anti–mouse α-IFNAR was purchased from Leinco and was used at concentrations ranging from 0.1 μg/mL to 10 μg/mL, as indicated (I-401, clone MAR1-5A3). Mouse isotype control antibody (mouse IgG1, Cell Signaling Technology, 5415) was used as a negative control for this antibody where applicable. Nec-1 was used to inhibit necroptosis; it was purchased from Selleckchem and used at 50 μM, unless otherwise specified (catalog S8037). Pan-caspase inhibitor Z-VAD-FMK was used at 10 μM to block apoptosis and was purchased from Selleckchem (catalog S7023). MG132 was used at 10 μM to inhibit proteasomal degradation of proteins and was purchased from Selleckchem (catalog S2619). Pharmacological inhibitors of the CREB/CBP/β-catenin system were purchased from Selleckchem (PRI-724, catalog S8262; KG-501, catalog S8409; and C646, catalog S7152) and Tocris Biosciences (666-15, catalog 5661). Purified mouse IFN-β was purchased from PBL Assay Science (catalog 12405-1). Urolithin A was purchased from Selleckchem (catalog S5312) and was used as an inducer of mitophagy. N-acetylcysteine was purchased from Selleckchem (catalog S1623) and used as an antioxidant.

### Western blot.

Cellular proteins were extracted using the following lysis buffer: 150 mM NaCl, 50 mM Tris-HCl (pH 7.4), 10% glycerol, 1% NP-40, 10 mM sodium fluoride, 1 mM sodium orthovanadate, 10 mM N-ethylmaleimide, and Halt Protease Inhibitor Cocktail (Thermo Fisher Scientific). Lysis was performed on ice with agitation for 30 minutes, followed by 30-minute centrifugation for 30 minutes at 16,000*g* at 4°C. Clarified lysates were then denatured at 95°C for 8 minutes in the presence of 4× LDS sample loading buffer (Invitrogen) and 5% β-mercaptoethanol (Bio-Rad), and they were separated by SDS-PAGE with NuPAGE 4%–12% Bis-Tris 1.5 mm 15-well gels (Thermo Fisher Scientific). Proteins were then transferred to nitrocellulose using iBlot2 system (Thermo Fisher Scientific), and membranes were blocked in 5% milk/TBS-T for 1 hour, followed by incubation with primary antibody overnight. After washes and incubation with respective horseradish peroxidase–conjugated secondary antibodies (Jackson ImmunoResearch Peroxidase AffiniPure goat anti–mouse IgG (H+L) [catalog 115-035-146] at 1:10,000 or Peroxidase AffiniPure goat anti–rabbit IgG (H+L) [catalog 111-035-003] at 1:20,000) for 1 hour, protein bands were visualized using SuperSignal West Femto substrate (Thermo Fisher Scientific) with Image-Quant LAS 4000 biomolecular imager (GE Healthcare Life Sciences).

### Immunopurification.

Immunopurification of proteins was performed using HCE cells cultured in 15 cm dishes. Cells were collected after specified times of infection and/or treatment, washed with PBS, scraped in cold PBS on ice, and transferred to conical tubes. Cells were centrifuged 800*g* for 5 minutes at 4°C; then, cellular proteins were extracted with lysis buffer: 150 mM NaCl, 50 mM Tris-HCl (pH 7.4), 10% glycerol, 1% NP-40, 10 mM sodium fluoride, 1 mM sodium orthovanadate, 10 mM N-ethylmaleimide, and Halt Protease Inhibitor Cocktail (Thermo Fisher Scientific). After 30 minutes of lysis with agitation at 4°C, lysates were centrifuged at 16,000*g* for 30 minutes at 4°C to pellet insoluble cellular debris. Immunopurification antibody ICP0 (Santa Cruz Biotechnology Inc., sc-53070) or isotype control (mouse IgG1, Cell Signaling Technology, 5415) was then added to clarified lysates and rotated for 16 hours at 4°C. Protein A/G Dynabeads (Thermo Fisher Scientific, 88802) were added, and samples were rotated for 1 hour at 4°C. Four washes with magnetic separation were performed with lysis buffer. Beads were finally resuspended in LDS buffer with 5% β-mercaptoethanol and denatured at 95°C for 8 minutes, and SDS-PAGE was performed.

### Quantitative PCR.

RNA was extracted from cultured cells using TRIzol (Thermo Fisher Scientific, 15596018), following the manufacturer’s protocol, and complementary DNA was produced using High Capacity cDNA Reverse Transcription kit (Invitrogen). Where applicable, corneal tissues were extracted from mice and incubated in 50 μL of 2 mg/mL collagenase D (MilliporeSigma, C0130) in PBS for 1 hour at 37°C. Tissues were then triturated with a pipette tip, resuspended in TRIzol, and extraction of RNA and cDNA were performed as above. Quantitative PCR (qPCR) was performed using Fast SYBR Green Master Mix (Invitrogen) on QuantStudio 7 Flex system (Invitrogen). To quantify viral genomes, infected cell pellets were resuspended in 500 μL virion buffer: 8 mg/mL Tris-HCl, 1% SDS, 10 mM EDTA supplemented with 1 μL proteinase K (Thermo Fisher Scientific, EO0491) and incubated at 55°C for 16 hours. Viral DNA was then extracted by adding 500 μL of Ultrapure phenol chloroform isoamyl alcohol mix (Thermo Fisher Scientific, 15593-031), according to the manufacturer’s specifications. The gD-specific primers listed below were then used to quantify HSV-1 genomes.

The following mouse-specific primers were used in this study: IFN-β forward (Fwd) 5′-TGTCCTCAACTGCTCTCCAC-3′, reverse (Rev) 5′-CATCCAGGCGTAGCTGTTGT-3′; ISG15 Fwd 5′-AGCAATGGCCTGGGACCTAAAG-3′, Rev 5′-CCGGCACACCAATCTTCTGG-3′; and β-actin Fwd 5′-GACGGCCAGGTCATCACTATTG-3′, Rev 5′-AGGAAGGCTGGAAAAGAGCC-3′. The following HSV-1–specific primers were used in this study: gD Fwd 5′- GTGTGACACTATCGTCCATAC-3′, Rev 5′-ATGACCGAACAACTCCCTAAC-3′ and ICP0 Fwd 5′-ACAGACCCCCAACACCTACA-3′, Rev 5′-GGGCGTGTCTCTGTGTATGA-3′

### Plaque assay.

Cell monolayers were inoculated for 2 hours in Opti-MEM (Thermo Fisher Scientific) with incubation at 37°C, 5% CO_2_; afterward, viral suspension was aspirated and replaced with complete media for the remaining infection time course. To quantify extracellular virus, culture supernatants were collected, centrifuged at 16,000*g* for 1 minute at room temperature, serially diluted in Opti-MEM, and overlaid on confluent monolayers of Vero cells in 24-well plates. After a 2-hour incubation, plaque inocula were aspirated, and cells were incubated with complete DMEM containing 0.5% methylcellulose (Thermo Fisher Scientific) for 48–72 hours. Medium was then aspirated, and cells were fixed with 100% methanol and finally stained with crystal violet solution to visualize plaques.

### Viral β-galactosidase entry assay.

Cell monolayers were inoculated with recombinant HSV-1 strain gL86, in which a portion of the gL gene was replaced with the lacZ gene encoding β-galactosidase. Only upon successful entry and expression of the viral genome is the β-galactosidase enzyme produced, and this virus is only capable of 1 round of viral replication; newly produced virions will not be capable of downstream infection. At 6 hours after infection, cells were washed once with PBS and incubated at 37°C for 1 hour with ONPG solution (Thermo Fisher Scientific, 34055; 3 mg/mL + 0.05% NP-40), and colorimetric substrate was detected at 410 nm using a microplate reader (Tecan GENious Pro).

### ROS detection assay.

WT and Hpse-KO MEFs were incubated with specified treatments for 24 hours, and cell culture supernatants were collected. ECL substrate SuperSignal West Pico (Pierce, 34080) reagents A and B were mixed in an equal ratio, and 50 μL was added to 100 μL of cell culture supernatant in flat-bottom 96-well plates. Photon counts were determined by Biotek Synergy H1 microplate reader.

### Immunofluorescence microscopy.

HCE cells or WT and Hpse-KO MEFs were cultured in glass-bottom dishes (MatTek Corporation) or 8-well μ-slides (iBidi). Cells were fixed in 4% paraformaldehyde for 10 minutes and permeabilized with 0.1% Triton-X (Thermo Fisher Scientific, BP151) for 10 minutes at room temperature for intracellular labeling. This was followed by incubation with primary antibody for 1 hour at room temperature. When a secondary antibody was needed, cells were incubated with respective FITC- or Alexa Fluor 647–conjugated secondary antibody (MilliporeSigma [F9137] or Thermo Fisher Scientific [A21244], respectively) at a dilution of 1:100 for 1 hour at room temperature. NucBlue Live ReadyProbes Hoechst stain (Thermo Fisher Scientific, R37605) was included with secondary antibody stains when applicable, according to manufacturer specifications. Samples were examined under LSM 710 confocal microscope (Zeiss) using a 63× oil immersion objective. For cell surface staining, cells were incubated with respective antibodies prior to fixation with paraformaldehyde for imaging. Fluorescence intensity of images was calculated using ZEN software.

### Live cell imaging.

WT and Hpse-KO MEFs were cultured in 24-well plates and infected with HSV-1 dual-color fluorescent virus expressing GFP driven by ICP0 promoter and RFP driven by gC promoter. At 2 hpi, inoculation media was replaced with complete DMEM, and cells were placed in the incubation chamber of Zeiss spinning disk live-cell imaging system, which maintains conditions of 37°C and 5% CO_2_. Images were captured on RFP, GFP, and bright-field at an interval of 30 minutes for 36 hours, and they were analyzed with ZEN software.

### Propidium iodide cell death assay.

Specified treatments were incubated with WT and Hpse-KO MEFs at 2 hpi in complete DMEM including propidium iodide (2 mg/mL) and Hoechst nucleic acid stain. At 24 hpi, Biotek Lionheart FX system was used to detect fluorescence intensity on PI, DAPI, and bright-field channels. Gen5 software was used to quantify positive events over 4 images per sample, and propidium iodide index was calculated as the quotient of PI^+^ events over DAPI^+^ events.

### Flow cytometry.

Corneas were extracted from mice after euthanasia and treated with 2 mg/mL collagenase D (MilliporeSigma, C0130) for 1 hour at 37°C and triturated with a pipette tip. Cell suspensions were filtered through a 70 μm mesh, resuspended in FACS buffer (PBS + 5% FBS), and counted by hemocytometer. In total, 1 × 10^6^ cells from each sample were aliquoted into U-bottom 96-well plates for subsequent staining. F_C_-receptors were blocked using TruStain fcX (BioLegend, 101319), and the following fluorophore-conjugated primary antibodies from BioLegend were used for cell surface staining: FITC anti–mouse CD45 (catalog 103107) and APC anti–mouse Ly-6G/Ly-6C (Gr-1, catalog 108411). Cells were immunolabeled, washed, and analyzed with Accuri C6 Plus flow cytometer (BD Biosciences). For flow cytometric quantification of cell death by PI uptake, cells were either infected with HSV-1 KOS (MOI of 0.1 or 1) or mock treated for 24 hours in medium containing PI. At the termination of cellular incubations, cells were collected on ice, washed twice with FACS buffer, and analyzed with Accuri C6 Plus flow cytometer. BD Accuri C6 Plus software and Treestar FlowJo v10.0.7 were used for all flow cytometry data analysis.

### Mouse tissue sectioning and staining.

For H&E staining, mice whole eyes were extracted after euthanasia, washed in PBS, embedded in Tissue-Tek OCT compound (Sakura Finetek), frozen on dry ice, and stored at –80°C. Sections (10 μm) were cut and adhered to Superfrost Plus glass slides (Thermo Fisher Scientific) using Cryostar NX50 microtome (Thermo Fisher Scientific), air dried at room temperature for 10 minutes, and then fixed in ice-cold acetone for 5 minutes. Slides were then incubated in the following series: 2 minutes running water, 1 minute Mayer’s hemalum solution (Merck HX73030749), 2 minutes running water, 2 minutes 70% ethanol, 1 minute 100% ethanol, 1 minute eosin Y solution with phloxine (MilliporeSigma, HT110316), 2 minutes 70% ethanol, 1 minute 100% ethanol, and 1 minute xylene. Then, slides were finally mounted and cover slipped with permount before they were imaged using Zeiss Axioskop 2 Plus microscope.

### RNA-seq.

WT and Hpse-KO MEFs were cultured in biological triplicates in 10 cm dishes with HSV-1 infection using strain KOS (MOI of 0.1) at time points of 0, 12, 24 and 36 hpi for a total of 24 samples. At specified time points, cells were washed once in PBS and collected by scraping on ice. In total, 1 × 10^6^ cells from each dish were used for RNA-seq workflow, and the remaining 4 × 10^6^ cells were used for quantitative Proteomics; the 2 analyses were performed on the same sets of cells. Cell pellets were suspended in RNAlater (Thermo Fisher Scientific, AM7020) and stored at –80°C until further processing. RNeasy Mini kit (Qiagen, 74104) was used to extract RNA, and DNA libraries for sequencing were constructed using Nextera XT DNA Library Prep kit (FC-131-1024, Illumina). The 24 prepared libraries were pooled and submitted to Michigan State University Genomics Facility (East Lansing, Michigan, USA), where they were quality checked using Qubit dsDNA HS, Agilent Bioanalyzer DNA 1000, and Kapa Illumina Library Quantification qPCR assays. The pool of libraries was loaded onto 1 lane of an Illumina HiSeq 4000 flow cell, and sequencing was performed in a 2 × 150 bp paired end format using HiSeq 4000 SBS reagents (Illumina); approximately 312 million acceptable quality reads were achieved. Base calling was done by Illumina Real Time Analysis v2.7.7, and output was demultiplexed and converted to FastQ format with Illumina Bcl2fastq v2.19.1. Assembly and alignment to Mus musculus (GRCm38.p6) and HSV-1 (JQ673480.1) genomes was performed with CLC Genomics Workbench. Subsequent analyses were performed in *R* v3.5. Differential expression analysis and normalization was performed with DESeq2, with design = ~ Genotype + Time.point + Genotype:Time.point and reduced model = ~ Genotype + Time.point, using likelihood relatedness test. Clustering of significantly differentially expressed genes (α = 0.01) was performed with Rtsne (15) and APcluster (16) based on temporal expression pattern. Transcription factor binding site enrichment analysis was performed using the PASTAA algorithm (52). Time series splines were generated with MetaLonDA (53). GO analysis was performed with ClueGO v2.5.2 (54) within Cytoscape v3.6.1 and ClusterProfiler v3.10.0 (55) in *R*.

### Mass spectrometry–based proteomic sample preparation.

Proteomic samples were processed as previously described with minor alterations (56). Cell pellets from HSV-1 infection time course were resuspended in 500 μL lysis buffer (75 mM NaCl, 3% SDS, 1 mM NaF, 1 mM β-glycerophosphate, 1 mM sodium orthovanadate, 10 mM sodium pyrophosphate, 1 mM PMSF, 50 mM HEPES [pH 8.5], and 1× Roche Complete EDTA-free protease inhibitor cocktail, followed by addition of 500 μL solution of 8M urea + 50 mM HEPES [pH 8.5], and solubilized by probe sonication [QSonica, Q500]). Samples were next subjected to reduction of disulfide bonds in 5 mM DTT for 30 minutes at 56°C. Free disulfide bonds were then alkylated for 20 minutes in the dark via the addition of iodoacetamide to a final concentration of 15 mM, followed by addition of the original volume of DTT and 15-minute incubation in the dark. One quarter volume of trichloroacetic acid was then added to precipitate proteins. Samples were incubated on ice for 10 minutes and then centrifuged for 5 minutes at 18,000*g* at 4°C. The supernatant was removed, and protein was washed twice via addition of ice-cold acetone, followed by centrifugation at 18,000*g* at 4°C for 2 minutes between each wash. Pellets were dried and resuspended in a solution of 1M urea + 50 mM HEPES (pH 8.5) for digestion, performed in 2 steps. First, samples were incubated overnight at room temperature on a shaker in the presence of 3 μg LysC Endopeptidase (VWR). Next, samples were incubated for 6 hours at 37°C with 3 μg Sequencing Grade Modified Trypsin (Promega). Digestion was terminated via addition of 20 μL trifluoroacetic acid (TFA). Samples were subjected to centrifugation at 18,000*g* at 4°C for 5 minutes to pellet insoluble debris; supernatants were desalted using C18 resin columns (Waters), and samples were dried under vacuum. Extracted peptides were resuspended in a solution of 50% acetonitrile + 5% formic acid (FA) and quantified using a Quantitative Colorimetric Peptide Assay (Pierce), as recommended by the manufacturer. In total, 50 μg of each sample was separated for subsequent sample preparation. A pooled “bridge channel” was made containing equal amounts from each sample, and two 50 μg aliquots were separated for inclusion in subsequent sample preparation steps. Aliquots (50 μg) were dried under vacuum. The 50 μg aliquots were next labeled using tandem mass tags (TMT) (Thermo Fisher Scientific, 90309). Dried peptides were resuspended in a solution of 30% dry acetonitrile (ACN) + 200 mM HEPES (pH 8.5). Label assignment was performed so that each biological replicate was represented within each 10-plex and such that no 2 replicates were assigned to the same label, except for the bridge channel internal standards. In total, 8 μL of 20 μg/μL TMT solution was added to the appropriate assigned sample, and the labeling reaction was allowed to proceed for 1 hour at room temperature. Reaction quenching was performed via addition of 9 μL 5% hydroxylamine at room temperature for 15 minutes. A total of 50 μL 1% TFA was added to each sample, and samples within each 10-plex experiment were multiplexed, desalted, and vacuum dried as described above. The 10-plex were fractionated using reverse phase high pH liquid chromatography. Samples were resuspended in 110 μL 5% ACN + 5% FA. Fractionation was performed on an Ultimate 3000 HPLC with 4.6 mm × 250 mm C18 column. Samples were separated on a 1-hour solvent gradient ranging from 5% to 90% ACN + 10 mM ammonium bicarbonate. Fractions were pooled as previously described and dried under a vacuum (57). Alternating pooled samples were used for subsequent analysis, except for the tenth pooled fraction, where its complement was used.

### Mass spectrometry–based proteomic analysis.

Dried fractions were resuspended in 8 μL 5% ACN + 5% FA and analyzed on an Orbitrap Fusion with in-line Easy Nano-LC 1000 (Thermo Fisher Scientific). Fractions were run as 3-hour gradients progressing from 3% ACN + 0.125% FA to 100% ACN + 0.125% FA. Fractions were loaded onto an in-house packed column of 0.5 cm C4 resin with 5 μm diameter, 0.5 cm C18 resin with 3 μm diameter, and 29 cm of C18 resin with 1.8 μm diameter. The column measured 30 cm in overall length, with an inner diameter of 100 μm and an outer diameter of 360 μm. Electrospray ionization was achieved at the source through application of 2000 V of electricity though a T-connector at the junction between the source, waste, and column capillaries. Spectra were acquired at the MS1 level in data-dependent mode with a scan range in the Orbitrap between 500 and 1200 *m/z* and resolution of 60,000. Automatic gain control (AGC) was set to 2 × 10^5^, with the maximum ion inject time set to 100 ms. The Top N setting was used for subsequent fragment ion analysis, with *n* = 10. MS2 data collection was performed using the decision tree option, wherein ions with 2 charges were analyzed between 600 and 1200 *m/z*. Ions with 3–4 charges were analyzed between 500 and 1200 *m/z*. The lower ion intensity threshold was set to 5 × 10^4^, and selected ions were isolated in the quadrupole at 0.5 Th. Fragmentation was performed using Collision-Induced Dissociation, and ion detection and centroiding occurred in the linear ion trap with AGC rapid scan rate of 1 × 10^4^. MS3 data were generated using synchronous precursor selection (58). No more than 10 precursor ions were concurrently fragmented using High Energy Collisional Dissociation fragmentation, and reporter ions were detected in the Orbitrap. The resolution was set to 60,000 with a lower threshold of 110 *m/z*. AGC of 1 × 10^5^ was used here, with a maximum inject time of 100 ms. All data were collected in centroid mode.

### Proteomic data analysis.

Spectral data were analyzed using Proteome Discoverer 2.1. Spectral matching was performed against a concatenated database of *Mus musculus* reference proteome appended to an HSV-1 reference proteome. SEQUEST-HT was used to match spectra against a target and decoy database generated in silico (59). Precursor mass and fragment ion mass tolerances were set to 50 ppm and 0.6 Da, respectively. Trypsin was specified as the enzyme, with 2 missed cleavages permitted. The length of accepted peptides was specified as 6–144 amino acids. Oxidation of methionine residues was specified as a variable modification (+15.995 Da); TMT labeling at N-termini and on lysine residues (+229.163 Da) and carbamidomethylation of cysteine residues (+57.021 Da) were specified as invariable modifications. FDR of 1% was used to filter spectra at the peptide and protein level. Data were processed by filtering peptide spectral matches (PSMs) to retain only high-quality and low ambiguity spectra. Any missing quantitation values within a 10-plex were replaced by a 1 to represent a baseline noise value. PSMs with average quantitation values less than 10 or isolation interference greater than 25 were removed from the data set. PSMs were summed at the protein level, and raw values were normalized against the median bridge channel value.

### Data and code availability.

Raw data for transcriptomics experiments were deposited as fastq files in the Sequence Read Archives (SRA) under BioProject PRJNA553809. Raw data for HSV-1 time course proteomics experiments can be found on ProteomeXchange under the identifier PXD014181. Hallmark_Interferon_Alpha_Response, GO NLRP3 Inflammasome Complex Assembly and GO Mitophagy gene sets were obtained from the publicly available GSEA/mSigDB collection at http://software.broadinstitute.org/gsea/msigdb/index.jsp Gene Expression Omnibus data set GSE34080 was accessed at https://www.ncbi.nlm.nih.gov/geo There are no restrictions on raw data; all requests can be addressed to the corresponding author.

### Statistics.

Statistical tests including unpaired, 2-tailed *t* test, Mann-Whitney *U* test, Wilcoxon signed-rank test, and 2-way ANOVA with correction for multiple comparisons were implemented in GraphPad Prism and *R* where appropriate, as indicated in the figure legends. Experiments were replicated 3 times unless otherwise specified. *P* < 0.05 was considered to be statistically significant. *P < 0.05, **P < 0.01, ***P < 0.001, ****P < 0.0001.

### Study approval.

All animal care and procedures were performed in accordance with the institutional and NIH guidelines and were approved by the Animal Care Committee at University of Illinois at Chicago (ACC protocol nos. 17-077 and 20-065).

## Author contributions

AA and DS conceptualized and designed the study. AA, BAT, RKS, TY, DJ, JA, JH, LK, CDP, SRH, EJK, AC, and JMW performed experiments and analyzed the data. AA and BAT performed data visualization. AA constructed figures and wrote the original manuscript. AA, BAT, RKS, TY, DJ, JA, JH, LK, CDP, SRH, EJK, AC, JMW, DJG, IV, JPL, DLP, PWF, and DS reviewed and edited the manuscript. AA, BAT, AC, JMW, DJG, IV, JPL, DLP, PWF, and DS provided unique resources for the project. AA, DJG, DLP, PWF, and DS supervised the study. AA and DS acquired funding.

## Supplementary Material

Supplemental data

Supplemental Data Set 1

Supplemental Data Set 2

Supplemental Data Set 3

Supplemental Data Set 4

Supplemental Data Set 5

## Version 1 02/23/2021

In-Press Preview

## Version 2 04/08/2021

Electronic publication
