# Supplementary material for: Disruption of innate defense responses by endoglycosidase HPSE promotes cell survival
Source: JCI Insight. 2021 Apr 8;6(7):e144255. doi: 10.1172/jci.insight.144255 (PMC8119219; doi:10.1172/jci.insight.144255)
Supplement: Supplemental data [file jciinsight-6-144255-s172.pdf]

Supplemental Material

Disruption of innate defense responses by endoglycosidase HPSE promotes cell survival

Alex Agelidis[1,2], Benjamin A. Turturice[1,3], Rahul K. Suryawanshi[2], Tejabhiram Yadavalli[2], Dinesh Jaishankar[2,4], Joshua Ames[1,2], James Hopkins[1,2], Lulia Koujah[1,2], Chandrashekhar D. Patil[2], Satvik R. Hadigal[2], Evan J. Kyzar[5], Anaamika Campeau[6,7], Jacob M. Wozniak[6,7], David J. Gonzalez[6,7], Israel Vlodavsky[8], Jin-ping Li[9], David L. Perkins[10,11], Patricia W. Finn[1,3], Deepak Shukla[1,2,*]

[1] Department of Microbiology and Immunology, University of Illinois at Chicago, Chicago, IL 60612, USA
[2] Department of Ophthalmology and Visual Sciences, University of Illinois at Chicago, 1855 West Taylor Street, MC 648, Chicago IL 60612, USA
[3] Division of Pulmonary, Critical Care, Sleep, and Allergy, Department of Medicine, University of Illinois at Chicago, Chicago IL, USA
[4] Department of Dermatology, Lurie Comprehensive Cancer Center, Northwestern University, Chicago IL, USA
[5] Department of Psychiatry, University of Illinois at Chicago, Chicago IL, USA
[6] Department of Pharmacology, University of California, San Diego, La Jolla CA, USA
[7] Skaggs School of Pharmacy, University of California, San Diego, La Jolla CA, USA
[8] Technion Integrated Cancer Center (TICC), Rappaport Faculty of Medicine, Technion, Haifa, Israel
[9] Department of Medical Biochemistry and Microbiology, University of Uppsala, Uppsala, Sweden
[10] Division of Nephrology, Department of Medicine, University of Illinois at Chicago, Chicago IL, USA
[11] Department of Surgery, University of Illinois at Chicago, Chicago IL, USA
[*] Correspondence addressed to: dshukla@uic.edu, (312) 996-0908

Figure S1 Viral replication is diminished in the absence of HPSE

Figure S2 Analysis of publicly available microarray dataset of HPSE knockdown in human cells yields similar results to analysis of HPSE knockout mouse cells

Figure S3 Demonstration of transcriptomics clustering method

Figure S4 Time series analysis of clusters of significantly differentially expressed genes in HPSE-deficient cells

Figure S5 Selective defect in viral protein production in Hpse-deficient cells

Figure S6 Quantification of corneal infiltration and flow gating strategy

Figure S7 Inverse association between CREB and IFN expression

Figure S8 Measurement of reactive oxygen species and cell death

Figure S9 Western blot analysis of protein markers of cell death

Figure S10 Full length western blots from specified figures

Dataset S1 Transcriptomics count matrices

Dataset S2 Baseline transcriptomics differential expression

Dataset S3 Results of clustered transcriptomics ClueGO gene ontology analysis

Dataset S4 Results of baseline transcriptomics ClueGO gene ontology analysis

Dataset S5 Quantitative proteomics normalized expression matrix

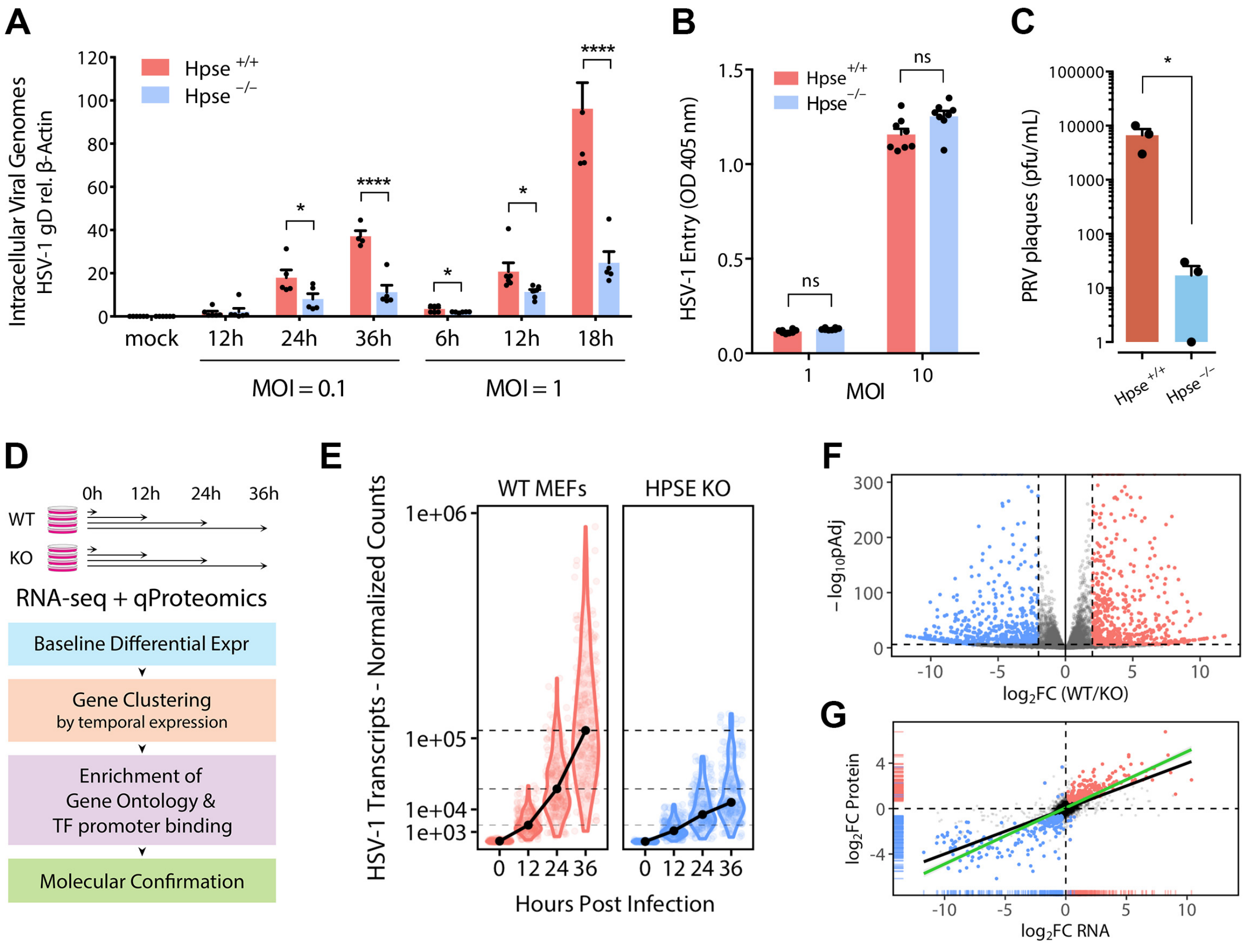

Figure S1 | Viral replication is diminished in the absence of HPSE. A, HSV-1 intracellular genome counts relative to β-actin in respective cell type measured by quantitative PCR (*n*=6). **B,** Viral entry at multiplicity of infection (MOI) of 1 and 10 quantified using β-galactosidase expressing HSV-1 (gL86 strain) and measurement of colorimetric substrate optical density (*n*=8). **C,** Plaque formation by pseudorabies virus (PRV) in wildtype and Hpse-deficient cells (*n*=3). **D,** Experimental design and analysis pipeline of unbiased approaches. **E,** HSV-1 viral gene expression as quantified by transcriptomics analysis. Black dots indicate mean expression for 74 detected viral transcripts. Dashed lines indicate median levels of gene expression in wildtype cells for comparison. **F,** Volcano plot depicting baseline differential expression in transcriptomic dataset, with red and blue points corresponding to significantly upregulated genes in Hpse $^{+/+}$ and Hpse $^{-/-}$ cells, respectively. **G,** Concordance analysis of baseline RNA-seq and proteomics datasets. Black dots and line indicate non-significantly differentially expressed genes, while colored dots and line represent significantly differentially expressed genes in both datasets. Data represent mean ± SEM. Significance determined by unpaired t-test with Holm-Sidak correction for multiple comparisons where applicable. *p<0.05, ****p<0.0001, ns, not significant.

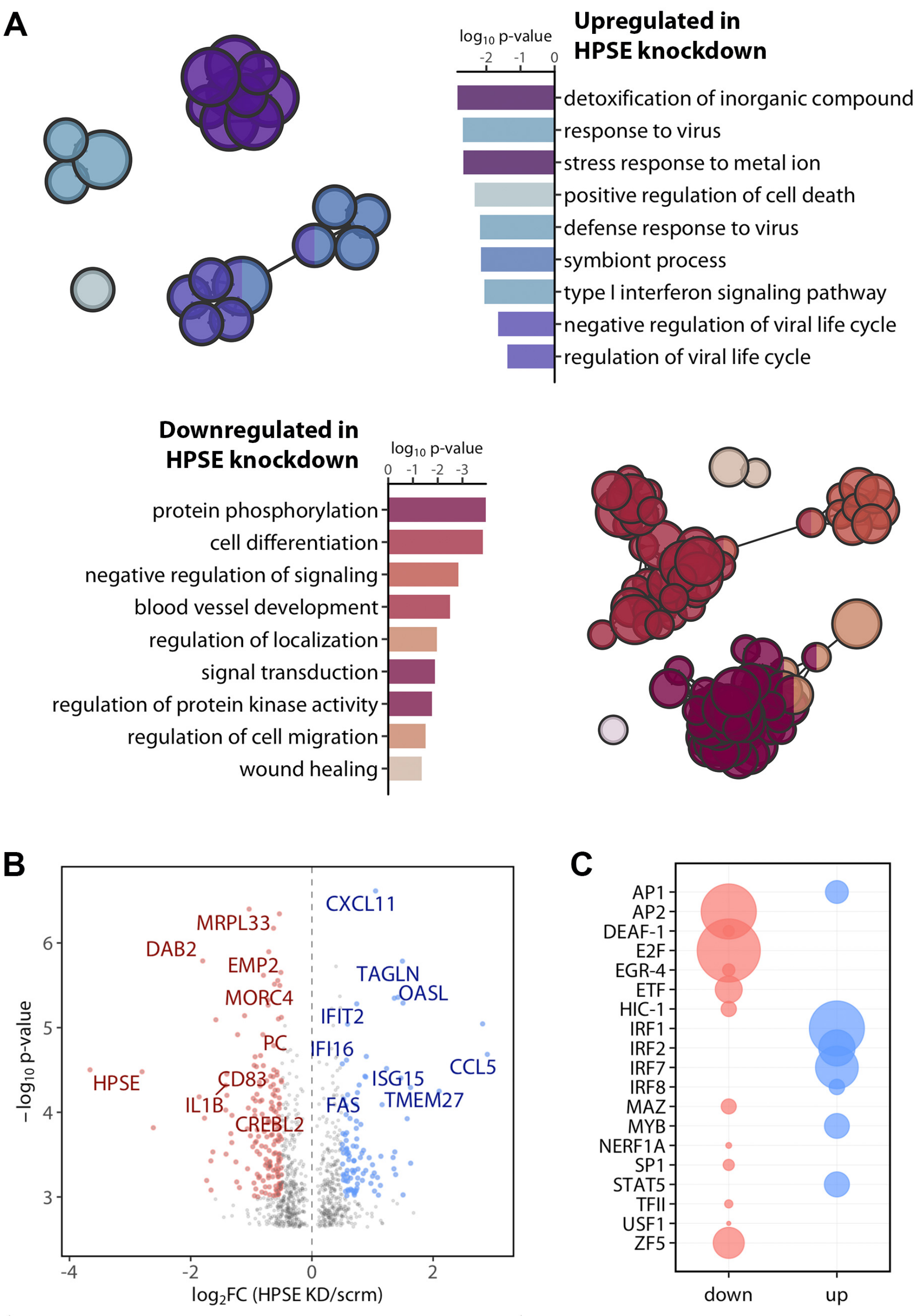

Figure S2 | Analysis of publicly available microarray dataset of HPSE knockdown in human cells yields similar results to analysis of HPSE knockout mouse cells. A, Gene ontology (GO) term enrichment analysis of HPSE siRNA knockdown of human gastric carcinoma cells (Gene Expression Omnibus dataset GSE34080), re-analyzed in our lab in R and ClueGO/Cytoscape. Each node represents a significantly enriched, with fold enrichment relative to node size and related terms grouped by color and spatial proximity. Each bar is color coded to match the node it represents. **B,** Volcano plot indicating gene up- and down-regulation of HPSE-depleted human cells. **C,** Transcription factor binding site enrichment analysis indicating major factors predicted using the PASTAA algorithm to regulate genes down- or up-regulated in this dataset.

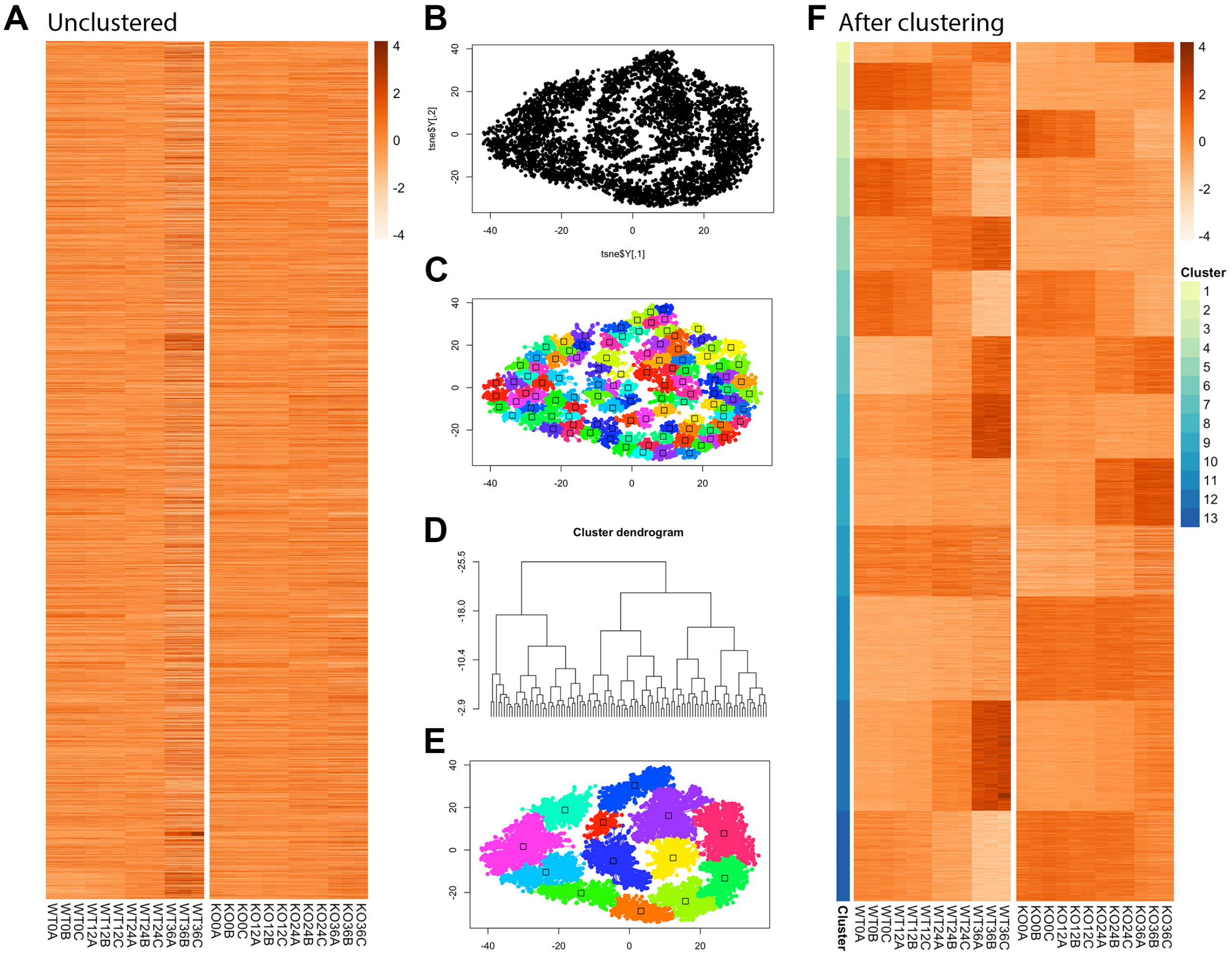

Figure S3 | Demonstration of transcriptomics clustering method. A, Heatmap of unclustered significantly differentially expressed genes (*n*=7015) shown in alphabetical order. **B-E,** Rtsne dimensional reduction and APcluster packages represent all genes in two dimensions and apply clustering based on temporal expression pattern. **F,** Heatmap of significantly differentially expressed genes represents 13 clearly defined clusters.

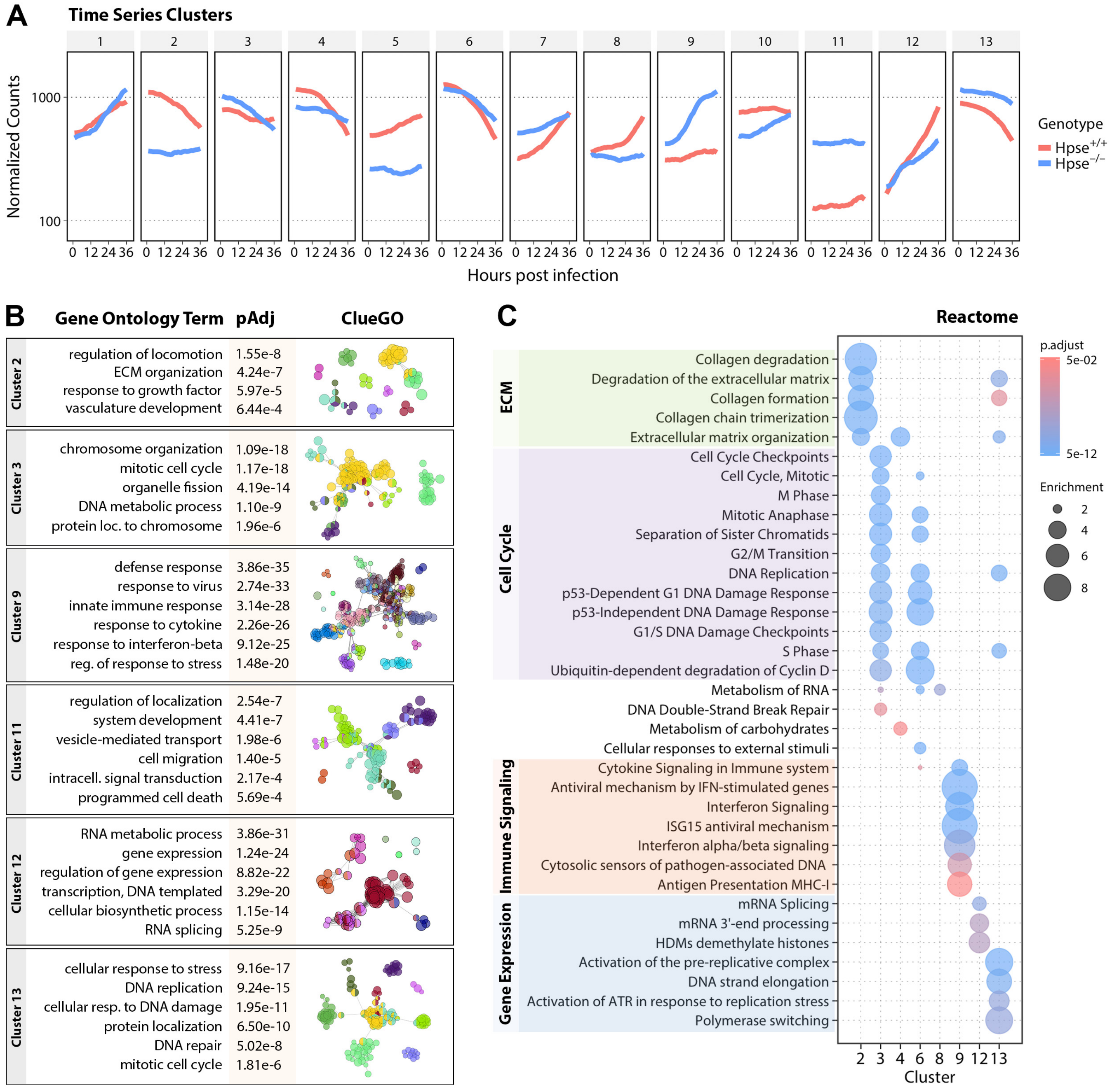

Figure S4 | Time series analysis of clusters of significantly differentially expressed genes in HPSE-deficient cells. A, Median gene expression pattern for each of 13 clusters at indicated time post HSV-1 infection. **B,** Selected gene ontology (GO) results for specified clusters with indicated significance values and diagram of connectedness at right generated with ClueGO/Cytoscape. Each node represents a separate GO term, with node size related to term enrichment and related terms grouped by color and spatial proximity. **C,** Graphical representation of Reactome analysis indicating major functional enrichments within each gene cluster.

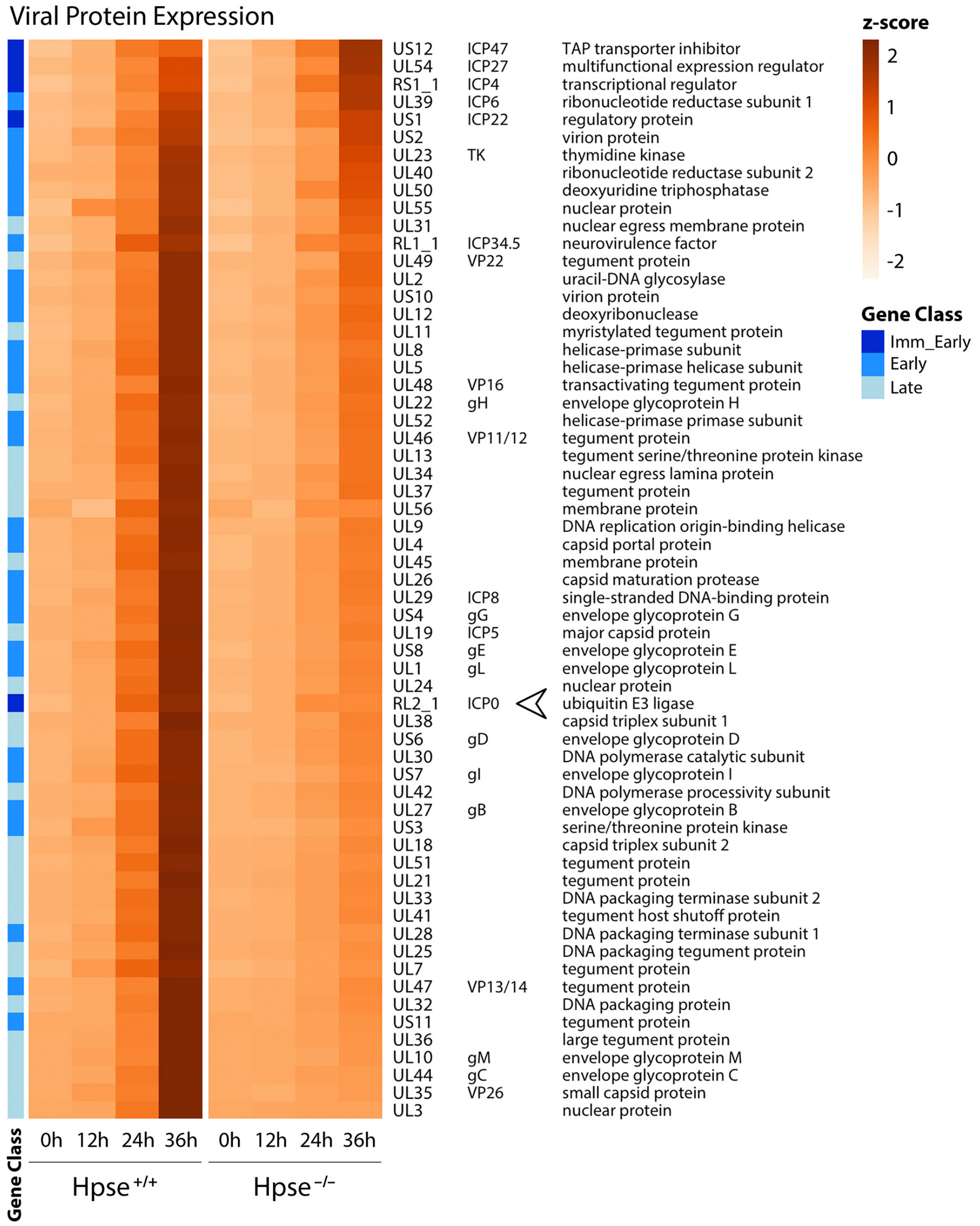

Figure S5 | Selective defect in viral protein production in Hpse-deficient cells. Heatmap of all viral proteins detected by quantitative tandem mass tag proteomics analysis. Arrowhead indicates ICP0 as the only immediate early viral protein with markedly diminished levels in Hpse-deficient cells.

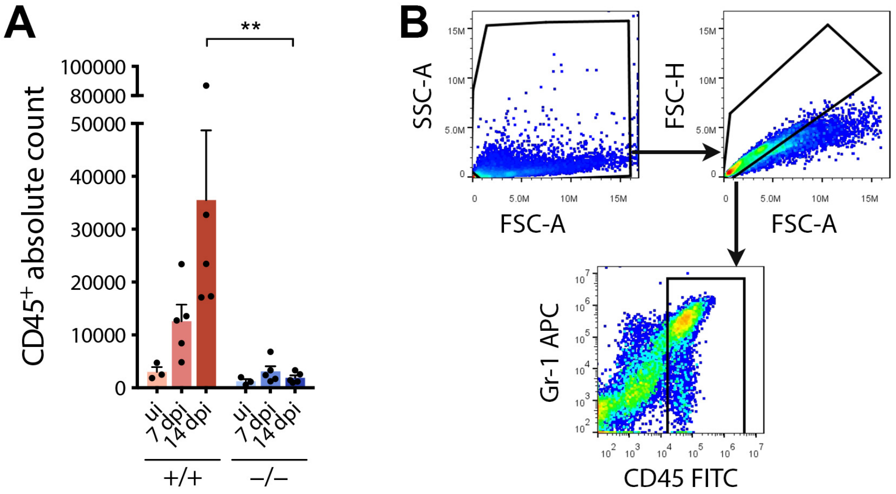

Figure S6 | Quantification of corneal infiltration and flow gating strategy. A, Total numbers of CD45-positive cells present in the corneas of Hpse-wildtype (+/+) and Hpse-deficient (–/–) mice at specified days post infection or uninfected (ui) (*n*=5 for infected animals, *n*=3 for uninfected animals). **B,** Flow gating strategy including non-debris singlets in the analysis. Data represent mean ± SEM. Significance determined by two-way ANOVA with Sidak correction for multiple comaparisons. **p<0.01.

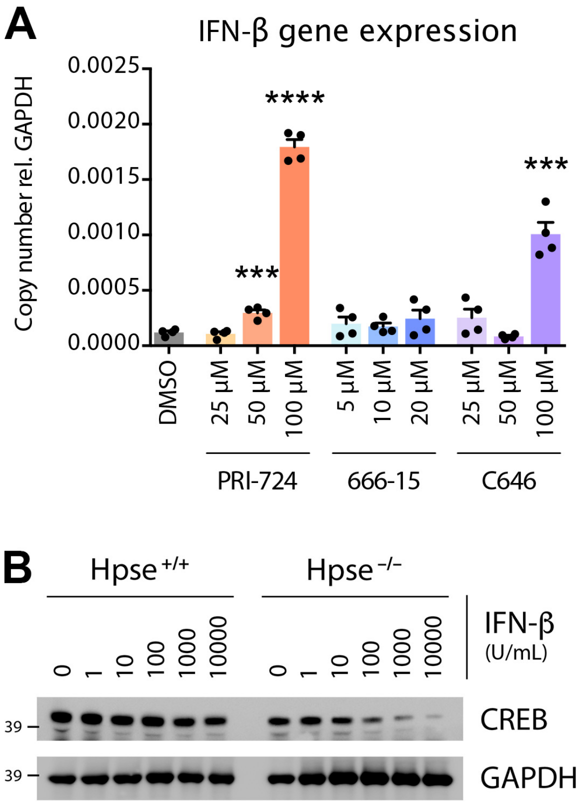

Figure S7 | Inverse association between CREB and IFN expression. A, IFN-β gene expression measured in human corneal epithelial (HCE) cells treated for 24 h with specified concentrations of inhibitors described in Figure 6. Significance determined by unpaired t-test with Holm-Sidak correction for multiple comparisons against DMSO control (*n*=4). **B,** Western blot analysis of CREB expression in Hpse-wildtype and Hpse-deficient MEFs after treatment with murine purified IFN-β for 16 h. Data represent mean ± SEM. ***p<0.001, ****p<0.0001.

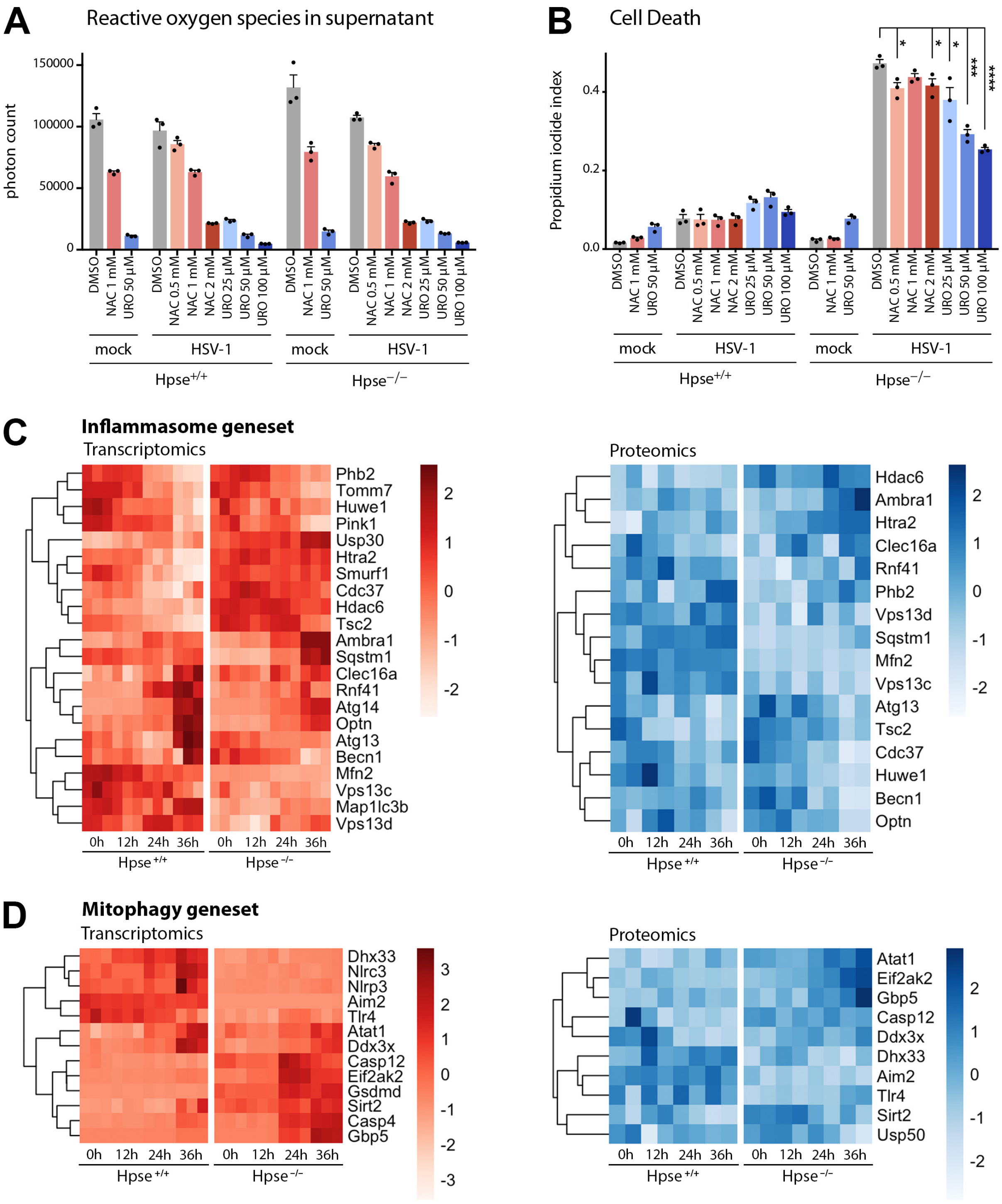

Figure S8 | Measurement of reactive oxygen species and cell death. A-B, Reactive oxygen species in supernatant (A) and cell death detected by propidium iodide (B) of MEFs treated at 2 hpi with N-acetylcysteine (NAC) and urolithin A (URO) and measured at 24 hpi. Significance determined by unpaired t-test against with Holm-Sidak correction for multiple comparisons against DMSO control (*n*=3). **C-D,** Subset analysis of transcriptomics (left) and proteomics (right) based on MSigDb genesets GO_NLRP3_INFLAMMASOME_ COMPLEX_ASSEMBLY (C) and GO_MITOPHAGY (D). Data represent mean ± SEM. *p<0.05, ***p<0.001, ****p<0.0001.

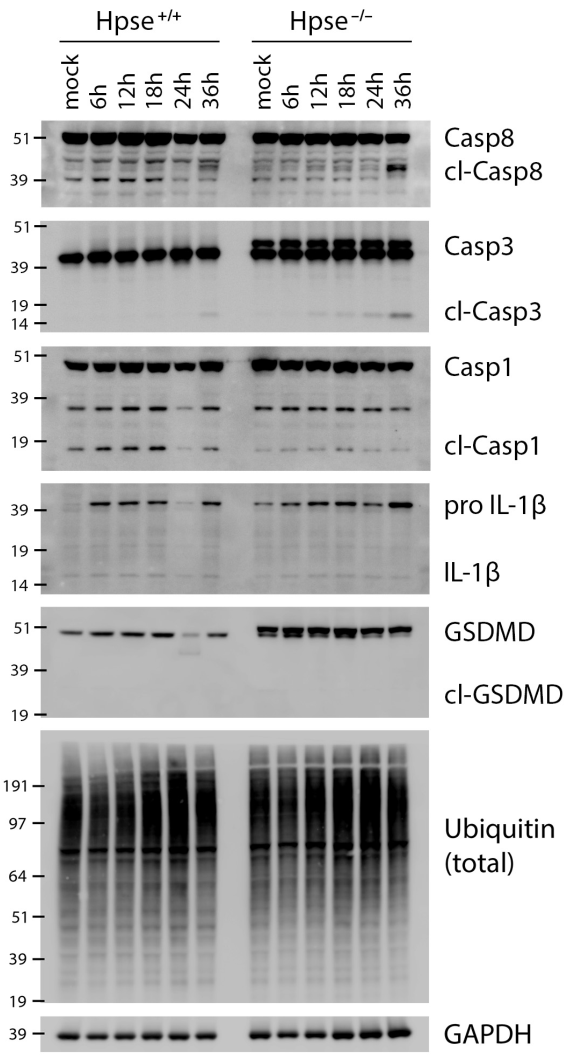

Figure S9 | Western blot analysis of protein markers of cell death. Caspase 8 and caspase 3 are classical representatives of apoptosis; caspase 1 and IL-1β signify inflammasome activation which can drive gasdermin D (GSDMD) cleavage and initiation of pyroptosis. Total protein ubiquitination was also analyzed and GAPDH is included as a protein loading control.

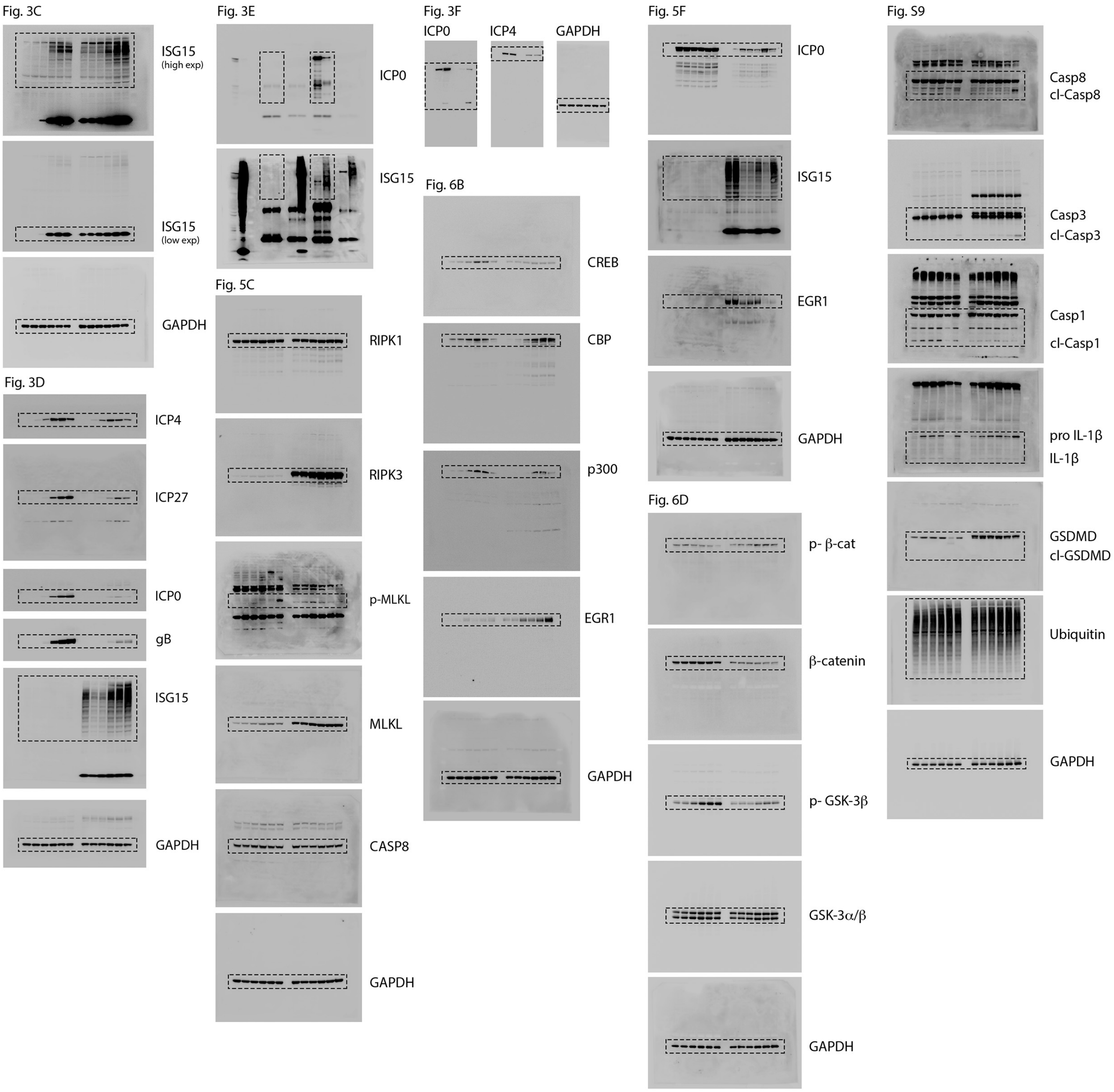

Figure S10 | Full length western blots from specified figures.
